# miRNA-like secondary structures in maize (*Zea mays*) genes and transposable elements correlate with small RNAs, methylation, and expression

**DOI:** 10.1101/gr.277459.122

**Published:** 2023-11

**Authors:** Galen T. Martin, Edwin Solares, Jeanelle Guadardo-Mendez, Aline Muyle, Alexandros Bousios, Brandon S. Gaut

**Affiliations:** 1 Department of Ecology and Evolutionary Biology, University of California, Irvine, California 92617, USA;; 2 Department of Ecology and Evolutionary Biology, University of California, Davis, California 95616, USA;; 3 CEFE, University of Montpellier, CNRS, EPHE, IRD, 34090 Montpellier, France;; 4 School of Life Sciences, University of Sussex, Brighton BN1 9QG, United Kingdom

## Abstract

RNA molecules carry information in their primary sequence and also their secondary structure. Secondary structure can confer important functional information, but it is also a signal for an RNAi-like host epigenetic response mediated by small RNAs (smRNAs). In this study, we used two bioinformatic methods to predict local secondary structures across features of the maize genome, focusing on small regions that had similar folding properties to pre-miRNA loci. We found miRNA-like secondary structures to be common in genes and most, but not all, superfamilies of RNA and DNA transposable elements (TEs). The miRNA-like regions map to a higher diversity of smRNAs than regions without miRNA-like structure, explaining up to 27% of variation in smRNA mapping for some TE superfamilies. This mapping bias is more pronounced among putatively autonomous TEs relative to nonautonomous TEs. Genome-wide, miRNA-like regions are also associated with elevated methylation levels, particularly in the CHH context. Among genes, those with miRNA-like secondary structure are 1.5-fold more highly expressed, on average, than other genes. However, these genes are also more variably expressed across the 26 nested association mapping founder lines, and this variability positively correlates with the number of mapping smRNAs. We conclude that local miRNA-like structures are a nearly ubiquitous feature of expressed regions of the maize genome, that they correlate with higher smRNA mapping and methylation, and that they may represent a trade-off between functional requirements and the potentially negative consequences of smRNA production.

In a highly simplified view, plant genomes consist of transposable elements (TEs) and genes. Both of these components use RNA to transmit coding information between one state (DNA) to another (protein). These RNA molecules carry information in their primary sequence of bases and also by their shape. This shape is primarily defined by the secondary structure of the transcript, a product of the intramolecular hydrogen bonds between RNA bases. Secondary structure can mediate the relationship between genotype and phenotype, because it affects the localization (Bullock et al. 2010), splicing (Buratti and Baralle 2004), and translation (Ding et al. 2014) of mRNAs. As a result, secondary structure influences nearly every processing step in the life cycle of transcripts (Vandivier et al. 2016).

Secondary structures can have another effect: They act as a template for small RNA (smRNA) production (Carthew and Sontheimer 2009; Li et al. 2012; Hung and Slotkin 2021). This production takes place through the binding of *Dicer-like* proteins (*DCL*s) (Axtell 2013; Fukudome and Fukuhara 2017) that degrade double-stranded RNA (dsRNA). In other words, when single-stranded RNA (ssRNA) forms a hairpin-like secondary structure, *DCL*s can recognize structured ssRNA as dsRNA and then degrade the dsRNA to produce smRNAs. This mechanism is essential for the biogenesis of microRNAs (miRNAs), a class of smRNAs that are generally ∼22 nt in length and that are derived from longer pre-miRNA transcripts with strong hairpin secondary structures (Carthew and Sontheimer 2009). However, this process is not limited to miRNAs, because 21- to 24-nt RNAs can also originate from the secondary structure of other non-miRNA transcripts (Slotkin et al. 2003; Li et al. 2012; Devert et al. 2015). These smRNAs can, in turn, cause transcripts to enter into the RNA interference (RNAi) pathway (Baulcombe 2004; Li et al. 2012; Cuerda-Gil and Slotkin 2016; Hung and Slotkin 2021). These observations suggest that sufficiently structured mRNAs, like miRNAs, form secondary structures that act as dsRNA substrates for degradation into smRNAs.

Little is known about how host genomes initially distinguish TEs from genes and target them for smRNA production (Marí-Ordóñez et al. 2013), but some studies suggest that hairpin structures in TE transcripts act as an immune signal for de novo silencing of certain TEs (Sijen and Plasterk 2003; Slotkin et al. 2003; Bousios et al. 2016; Hung and Slotkin 2021). One such example is *Mu-killer*, a locus that generates smRNAs and thereby silences *MuDR* elements (a DNA transposon) in maize (*Zea mays* ssp. *mays*) (Slotkin et al. 2003). *Mu-killer* consists of a truncated, duplicated, and inverted copy of *MuDR* that, when transcribed, creates a hairpin secondary structure and is subsequently cut into *trans*-acting small-interfering RNAs (siRNAs) that target active *MuDR* transcripts. Another potential example comes from Sirevirus long terminal repeat (LTR) retrotransposons in maize (Bousios et al. 2016), which occupy 21% of the maize B73 genome (Bousios et al. 2012). The investigators mapped smRNAs to full-length Sirevirus copies, reasoning that loci important for host-plant recognition and silencing should be associated with a larger number of smRNA sequences than other regions of the elements. An excess of smRNAs mapped to regions that had strong predicted secondary structure owing to clusters of palindromic motifs (Bousios et al. 2016). These studies present evidence that secondary structure helps initiate silencing of some TEs. In fact, one review has argued that the only characterized pathway to de novo smRNA production relies on RNA secondary structure (Hung and Slotkin 2021). It should be noted, however, that some phased siRNAs are caused by miRNA cleavage events that apparently do not require secondary structure (Creasey et al. 2014).

If RNA sequences form miRNA-like hairpin structures, leading to the production of smRNAs, two important questions must be addressed. First, how common are miRNA-like secondary structures across the immense diversity of plant TEs? One prominent review of smRNAs argued that there is an urgent need to annotate hairpins that may have the capacity to act as a template for smRNA production (Axtell 2013), but this need has not yet been met. Thus far, the importance of hairpin structure for de novo silencing has been implicated only in a few individual TE families. Second, secondary structure is not unique to TEs and exists within genes too. How often do genes have such structure, and is there evidence that genes form dsRNA substrates in these regions, too? Li et al. (2012) documented a positive relationship between stability of mRNA structure and smRNA abundance for *Arabidopsis thaliana* genes, suggesting that genic transcripts do form dsRNA substrates. Yet these genes are still expressed, potentially because of countermeasures that moderate the potential effects of smRNAs on genes, including hypothesized protection against RNAi caused by high GC content (Hung and Slotkin 2021) and active gene demethylation (Gong et al. 2002; Zhang et al. 2022). Although it has long been thought that miRNA loci may be derived from TE sequences (Smalheiser and Torvik 2005), there has not yet been, to our knowledge, a genome-wide comparison of miRNA-like secondary structures among genes and TE superfamilies.

In this study, we predict secondary structures in genes and TEs of the maize B73 genome. Secondary structure can be empirically measured through sequencing techniques such as DMS-seq and SHAPE-seq (Yang et al. 2018), which is applied to the transcribed component of whole genomes (Ding et al. 2014; Ferrero-Serrano et al. 2022). However, this approach requires that the sequences of interest are expressed, preventing comprehensive investigation of plant TEs, most of which are silent. These methods are also difficult to perform on large genomes with high repeat content, so that genome-wide “structurome” sequencing has thus far only been completed on plants with relatively small genomes, like *A. thaliana* (Ding et al. 2014; Bevilacqua et al. 2016) and rice, *Oryza sativa* (Ritchey et al. 2017). The second approach, which we adopted here, relies on bioinformatic predictions based on genome sequence data. Secondary structure prediction is a subject of active research, and methods vary in their predictions and accuracy. Here we use two separate methods that rely on distinct algorithms to identify regions with properties similar to miRNA-like hairpins. Briefly, the first uses RNAfold (Lorenz et al. 2011), which estimates the minimum free energy (MFE) of the most likely secondary structure of a given sequence (Nussinov and Jacobson 1980; Zuker and Stiegler 1981). Following precedence, we apply RNAfold in a windows-based approach. The second relies on a newer tool, LinearPartition (Zhang et al. 2020), that calculates a partition function for a complete (i.e., not windows-based) RNA sequence. The LinearPartition function includes the sum of equilibrium constants for all possible secondary structures for a sequence (i.e., not just the most likely structure). We focus specifically on detecting regions with miRNA-like secondary structures, because miRNAs are known to fold and thereby act as a dsRNA substrate for *Dicer*-like mechanisms.

After performing computational annotation to predict miRNA-like regions in the genes and TEs of maize, we investigate the relationship between these regions to smRNAs, methylation levels, chromatin accessibility, and, where applicable, gene expression (Supplemental Fig. S1). With these data, we address four sets of questions. The first focuses on predicted secondary structure: How often do TEs and genes contain regions of miRNA-like regions? Are these regions in specific locations? The second set of questions focuses on the relationship between secondary structure and smRNAs. Do miRNA-like regions consistently map more smRNAs, and, if so, of what size? The question of size is important because it is thought that dsRNA degradation via *Dicer* feeds into post-transcriptional gene silencing (PTGS) pathways, which tends to rely on 21- and 22-nt smRNAs. In contrast, pathways that lead to transcriptional gene silencing (TGS) tend to rely more often on 24-nt smRNAs, although these size distinctions are neither strict nor universal (Fultz and Slotkin 2017; Panda et al. 2020). Our third set of questions focuses on the potential genomic implications of hairpins and smRNAs. Do these miRNA-like regions have higher methylation levels or specific chromatin properties? Finally, we assess the effects of miRNA-like secondary structures on gene expression by including data from 26 parents of the maize nested association mapping (NAM) lines (McMullen et al. 2009; Hufford et al. 2021).

## Results

### Two methods to predict miRNA-like secondary structures and their comparison

**Figure 1.**
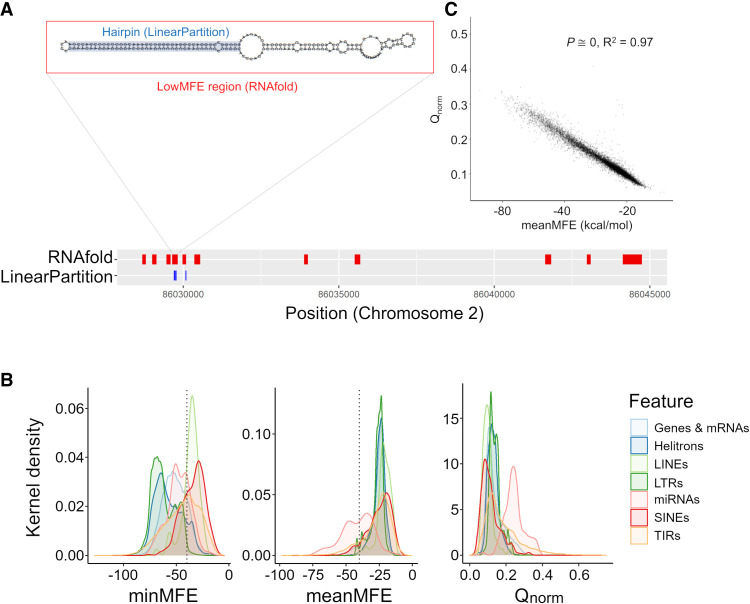
Characteristics of miRNA-like secondary structures across two methods. (*A*) A schematic contrasting the two prediction methods for a genic region on Chromosome 2. The LinearPartition (LP) method focuses on identifying small regions with hairpin characteristics, and the RNAfold method focuses on regions with low minimum free energy (MFE). This example illustrates lowMFE regions in red, with overlapping LP-hairpins in blue. Note that lowMFE regions exceed 110 bp, because they represent the concatenation of overlapping windows with MFE < −40 kcal/mol. (*B*) The correlation between meanMFE and *Q*_*norm*_ based on 39,179 genes. (*C*) The distributions of three summary statistics—minMFE, meanMFE, and *Q*_*norm*_—across seven feature categories. In the key, helitrons correspond to DHH elements (for the three letter designations, see Table 2); LTRs consist of RLC, RLG, and RLX; LINEs are the RIL and RIT elements; SINEs are RST; and terminal repeat elements consist of DTA, DTC, DTH, DTM, and DTT elements.

We adopted two complementary bioinformatic methods to identify miRNA-like hairpin regions (Fig. 1A). The details of their implementation are given in the Methods. Here, we provide an overview of the methods and compare their performance. To aid the reader, we also provide terms that are used to characterize analyzed sequences (Table 1).

**Table 1.**
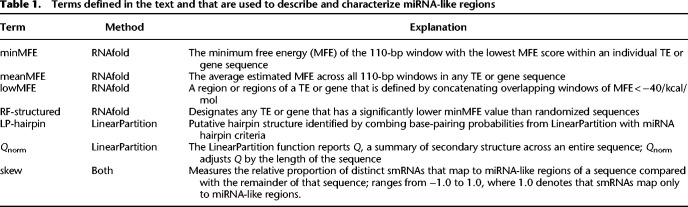
Terms defined in the text and that are used to describe and characterize miRNA-like regions

#### RNAfold

The first method applied RNAfold to sliding windows of 110 nt, following the methods of previous work (Wang et al. 2009; Bousios et al. 2016). The 110-nt windows were originally designed by Wang et al. (2009) to include regions that map 20- to 25-nt smRNAs, along with ∼90 bp of flanking sequence (Wang et al. 2009). This approach established that pre-miRNA windows of this size typically have MFEs < −40 kcal/mol (Wang et al. 2009); we used that empirical cutoff to define windows of secondary structure with miRNA-like stability. By focusing on regions of similar size to pre-miRNA transcripts and by using their empirical threshold cutoff of −40 kcal/mol, we, in effect, used miRNA loci as a “positive control” for ssRNAs that are expected to form secondary structures.

We applied RNAfold across features of the B73 reference maize genome (version 4.0) (Jiao et al. 2017). The features included miRNA precursor loci, TEs, and genes. The TEs included all families annotated by Jiao et al. (2017), including LTRs, terminal inverted repeat (TIR) elements, helitrons, long interspersed nuclear elements (LINEs), and short interspersed nuclear elements (SINEs). Within these TE types, we focused on superfamily categories (Wicker et al. 2007), which distinguished (e.g.,) between *Ty3*/RLG and *Copia*/RLC LTR elements and among TIR elements like *Mutators*/DTM and *Harbingers*/DTH. Note that throughout the paper, we refer to TE superfamilies by their names and also their three-letter designation from Wicker et al. (2007; Table 2). Notably, these annotations do not typically include miniature inverted terminal repeats (MITEs), a class of small nonautonomous TEs that often contain strong secondary structures. For genes, we studied both the annotated gene—which included untranslated regions (UTRs), exons, and introns—and mature transcripts that lacked introns. Altogether, with this method we examined 373,485 features representing 15 distinct feature categories (Table 2). Because we used sliding windows, each nucleotide within a feature corresponded to one sliding window (for all but the final 109 nt of a sequence). This approach was a massive bioinformatic undertaking, requiring an MFE calculation for a total of 3.56 billion windows.

**Table 2.**
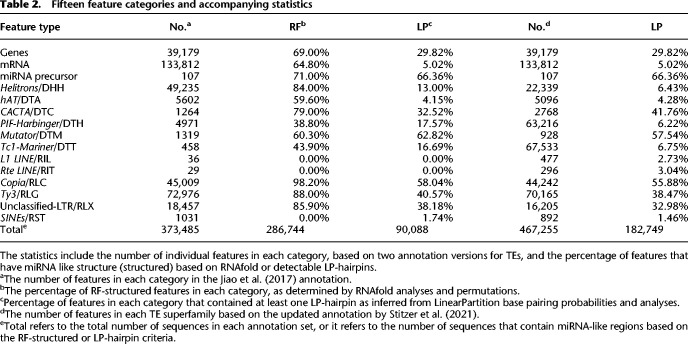
Fifteen feature categories and accompanying statistics

Because each feature consisted of many RNAfold windows, we used summary statistics to characterize local secondary structure in each feature (Table 1). These included the minimum MFE (minMFE), which was the MFE of the window with the strongest predicted secondary structure for each feature, and mean MFE (meanMFE), which averaged MFE across windows within a feature. For each feature, we also concatenated overlapping windows with MFE < −40 kcal/mol, designating these as lowMFE regions (Table 1; Fig. 1A,B).

One concern about using MFE as a quantitative statistic is that it varies by G:C composition (e.g., higher G:C content tends to induce more stable secondary structures) and primary sequence (e.g., whether the order of bases forms palindromes and stem-loop structures). Because we were primarily interested in secondary structure resulting from the latter, we controlled for base composition by randomizing the sequence of each feature five times and then repeating MFE predictions each time, requiring another 17.8 billion (=5 × 3.56 billion) window computations. By randomizing, we identified features that had more stable secondary structures than expected given their nucleotide composition. We then classified a feature as “RF-structured” (RF for RNAfold) when it contained windows with MFEs < −40 kcal/mol and also had a minMFE significantly lower than permutations (*P* < 0.05, one-sided Wilcoxon test, Benjamini and Hochberg corrected) (Table 1). Conversely, we labeled features as “unstructured” when their minMFE was not significantly lower than that of randomized sequences. Supplemental Figure S2 reports differences between randomized and observed minMFE values for each feature category; overall, 76% (286,774 of 373,485) of features were RF-structured (Table 2).

#### LinearPartition

The second prediction method was based on LinearPartition (Zhang et al. 2020). This approach did not rely on sliding windows to infer local secondary structure but analyzed the complete sequence of each feature. The advantage was that each feature required only one computational analysis, vastly improving computational burden and speed. Accordingly, we applied this method to the same set of 373,485 features as RNAfold but also to a larger, updated version of maize TE annotations (Stitzer et al. 2021), resulting in an expanded data set of 467,255 features (Table 2).

For each sequence, LinearPartition calculated the partition function, summarized by the parameter *Q*. For each nucleotide site within a feature, the method calculated a pairing probability between all nucleotides in the feature. We focused on nucleotide pairs with high probabilities of pairing (>0.90) and searched within each feature for runs of nucleotides that matched widely accepted miRNA annotation guidelines for plants (Axtell and Meyers 2018). These guidelines defined hairpins consisting of consecutive stretches of ≥21 nt that were likely to pair (>90% probability) with fewer than five mismatched nucleotides, including fewer than three mismatches in putative asymmetric bulges (i.e., places where the gap on one side of a hairpin was greater than the gap on the other side of the hairpin; Methods) (Fig. 1A). We called sequences that fit these criteria “LP-hairpins” (Table 1).

#### Comparing the methods

Both methods were designed to identify regions of strong local secondary structures within features, but they focused on different miRNA-like properties. Yet, they did yield significant consistencies and overlaps. For example, we contrasted the two entire-sequence summary statistics: meanMFE and the partition function normalized for feature length (*Q*_*norm*_). Across structured features, *Q*_*norm*_ correlated strongly with meanMFE (R^2^ = 0.73 across all feature types and R^2^ = 0.97 across genes; *P* = 0) (Fig. 1C) and weakly (R^2^ = 0.04) but still significantly (*P* = 3.05 × 10^−10^) with minMFE. We also compared the overlap in genomic locations between LP-hairpins and low (<−40) MFE regions (Fig. 1A). Across all of the 287,744 RF-structured features (Table 2), 78.46% of LinearPartition hairpins were within a lowMFE region. Given that lowMFE regions collectively comprised ∼22.95% of annotated features, this represented a substantial 12.2-fold enrichment of LP-hairpins within lowMFE regions. By design, lowMFE regions were much larger (median = 348 nt) than LP-hairpins (median = 25 nt) and therefore took up a much larger proportion of the space inside of comparable features. In total, lowMFE regions constituted 1.9 × 10^8^ nt versus 1.7 × 10^7^ nt for LP-hairpins.

### The prevalence and locations of miRNA-like secondary structures

#### Prevalence of miRNA-like secondary structures across TE superfamilies

Using both methods of prediction, we detected substantial variation in the prevalence of miRNA-like secondary structures among TE categories. Some TE superfamilies contained little evidence of structure. For example, the *LINE* (RIL and RIT) elements had no RF-structured elements and also had no detectable LP-hairpins (Table 2). Because the 2017 annotation from Jiao et al. (2017) contained few (*n* = 65) RIL and RIT elements, we repeated the LinearPartition analysis with an expanded set of *n* = 773 elements from Stitzer et al. (2021), finding again that only a small subset (∼3%) contained hairpins (Table 2). *SINEs*/RST also had very low incidences of miRNA-like structure, with no RF-structured elements and <2% containing LP-hairpins (Fig. 1B). In contrast to LINEs and SINEs, LTR elements generally had abundant miRNA-like structures. For example, 98% of *Copia*/RLC elements had RF-structure and 58.0% had LP-hairpins (Table 2; Fig. 1B). We note, however, that LTR elements were longer on average than the other TE subfamilies and also that there was an overall negative relationship between feature length and minMFE across all 15 feature categories (*P* < 2.2 × 10^−16^, R^2^ = 0.20, linear model) (Supplemental Fig. S3).

The prevalence of miRNA-like regions also varied among TIR superfamilies. *Mutator*/DTM elements were especially notable for the high percentage of elements with LP-hairpins (62.82%), whereas only 32.52% of *CACTA*/DTC elements contained LP-hairpins. Fewer than half of the annotated *Tc1-Mariner*/DTT and *PIF-Harbinger*/DTH elements were RF-structured or contained LP-hairpins (Table 2), but this corresponded to thousands of elements in these superfamilies.

It is worth making two overarching observations from the analyses reported in Table 2. First, the percentage of sequences identified by RNAfold and LinearPartition was correlated across the 15 feature categories (R^2^= 0.65; *P* < 0.001), suggesting again that the two methods identified similar characteristics in most superfamilies. Second, the expanded TE data set of Stitzer et al. (2021) showed similar trends to the Jiao et al. (2017) annotation data set (R^2^= 0.96; *P* < 0.001). For example, LINEs, SINEs, and *hAT*/DTA elements generally had low proportions of elements with LP-hairpins in both annotation sets, whereas LTR superfamilies had high proportions in both annotation sets.

#### Biases in the locations of miRNA-like regions

**Figure 2.**
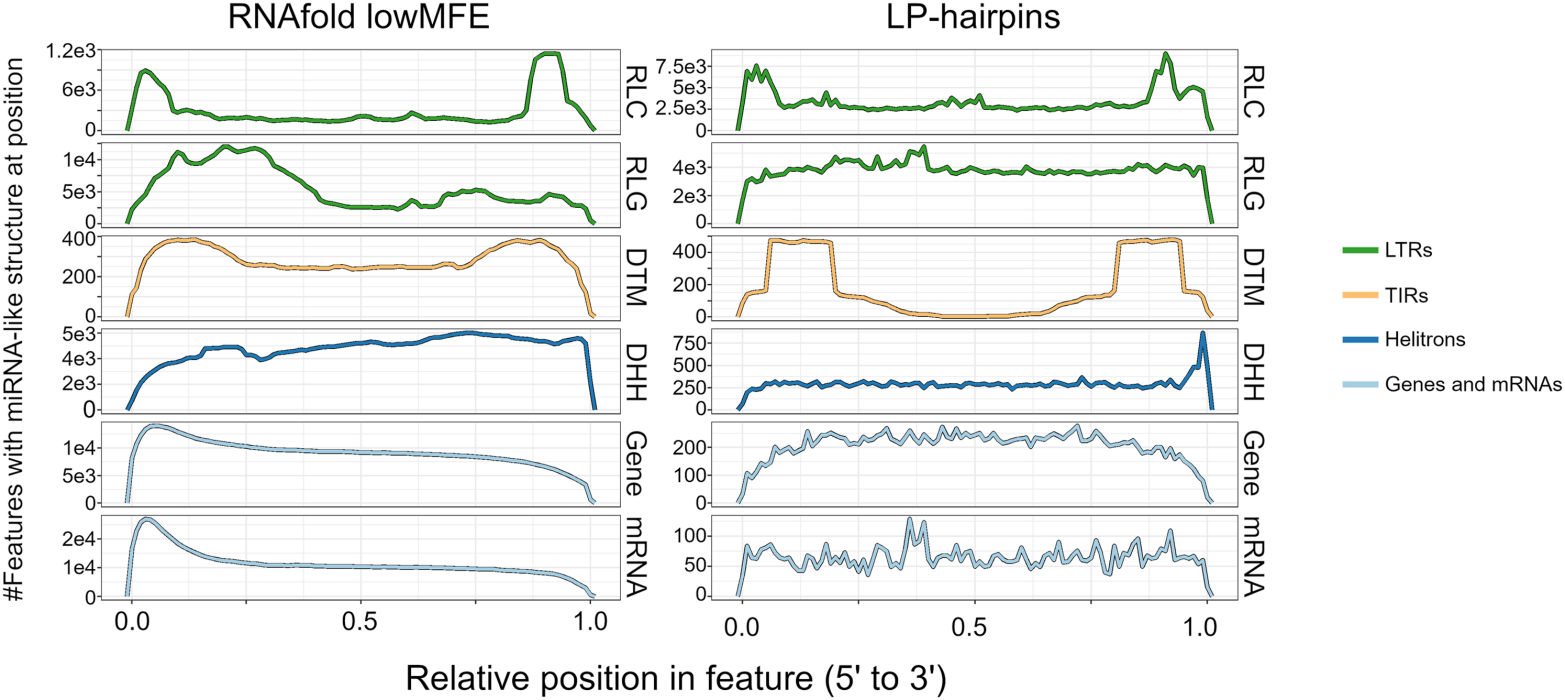
Landscapes of miRNA-like regions across feature types. Each row represents a metaprofile that combines data from all members of each feature type, based on structured members. Features were divided into 100 equally sized bins from the 5′ end to the 3′ end. The *left* column shows the number of features with lowMFE (<−40 kcal/mol) windows, and the *right* column shows the number of features with LP hairpins. A peak in the landscape represents a region that commonly contains miRNA-like structures. All panels share the same *x*-axis, which is represented proportionally across the length of features, from 0.00 (5′ end) to 1.00 (3′ end). This figure shows these locations for a subset of the 15 categories in Table 2; the remainder of the categories are shown in Supplemental Figure S4.

We next examined the locations of miRNA-like secondary structure across the length of each feature type. For these analyses, we focused only on the 286,744 features that were predicted to have RF-structure (Table 2). For each feature category, we separately mapped the positions of lowMFE regions and LP-hairpins along their lengths (Fig. 2). Consistent with previous work (Bousios et al. 2016), both lowMFE and LP-hairpins were concentrated within the LTRs of *Copia*/RLC elements. In contrast, *Ty3*/RLG elements generally lacked an obvious peak for miRNA-like structures. Most DNA transposon superfamilies had relatively uniform distributions of lowMFE regions across their lengths (Supplemental Fig. S4), but LP-hairpins were biased heavily toward the terminal inverted repeats for TIR elements like *Mutator*/DTM (Fig. 2), *hAT*/DTA, and *CACTA*/DTC elements (Supplemental Fig. S4). Finally, *Helitrons*/DHH had a distinct 3′ bias for both lowMFE regions and LP-hairpins (Fig. 2), reflecting the ∼11-nt stem-loop structure common to *Helitron* 3′ ends (Kapitonov and Jurka 2007; Xiong et al. 2014). Across TE superfamilies, some secondary structures had similar underlying sequences motifs. The most abundant consensus sequence of *Copia*/RLC elements was CACCGGACNNNGTCCGGTG, as reported previously (Bousios et al. 2016), which was present in 42.9% of RLC structured regions. This same palindrome was also the most abundant motif in *Helitron*/DHH transposons (MEME e-value = 1.0 × 10^−165^), appearing in 5231 DHH structured regions (10.7%) (Supplemental Fig. S5).

#### miRNA-like secondary structures within genes

A higher percentage (69.0%) of genes were RF-structured than contained LP-hairpins (29.8%) (Table 2). When we examined the distributions of miRNA-like structures across genes and their mature transcripts, we found that the two methods differed in their predictions. In 85% of genes (Fig. 2), lowMFE regions overlapped the 5′ UTRs, where secondary structures are known to participate in ribosome binding and translation (Babendure et al. 2006; Matoulkova et al. 2012). In contrast, LP-hairpins were fairly uniformly distributed across gene lengths (Fig. 2), with perhaps a slight bias toward the middle of the gene as documented previously in *Arabidopsis* (Li et al. 2012). Most (76.19%) of these LP-hairpins were found in introns, so that far fewer (5.02%) of mature mRNA transcripts had LP-hairpins (Table 2). These results show that 5′ UTRs commonly have regions of local secondary structure but infrequently contain LP-hairpins.

### Comparing miRNA-like regions to smRNA diversity

#### Correlations between miRNA-like regions and smRNA mapping abundance

Under the dsRNA-substrate model, genomic regions of high secondary structure should have homology with more smRNAs compared with nonstructured regions. To test the hypothesis, we mapped 21-, 22-, and 24-nt smRNAs from up to 42 published smRNA libraries (see Methods; Supplemental Table S1) to the B73 maize genome and then counted the number of distinct smRNA sequences (also known as “smRNA species”) (Bousios et al. 2017) that mapped with 100% identity to genomic regions. Because of their different functions (Axtell 2013; Borges and Martienssen 2015), we examined smRNAs in the three size classes (21, 22, and 24 nt) separately. Two caveats should be mentioned about these smRNAs: (1) Although many of these smRNAs may be hairpin-derived RNAs (hpRNAs) (Axtell 2013), we do not know their origin and refer to them by the more general “smRNA” term for clarity, and (2) we do not know that each smRNAs identified here functions as siRNA, merely that they are the correct size to act as a canonical siRNAs.

We first examined the relationship between miRNA-like regions and smRNAs using a linear model across all 373,485 features of the Jiao et al. (2017) annotation set, using correlation statistics. The correlation coefficient was generally small—for example, R^2^ was ∼0.1 for models incorporating minMFE—but highly significant (Table 3). Moreover, the results were significantly positive for all RNAfold and LinearPartition summary metrics (Table 3). Extending this approach separately to the 15 individual feature categories, three smRNA lengths, and three metrics (minMFE, meanMFE, and *Q*_*norm*_), 82% of correlations were significant after false-discovery rate (FDR) correction (Supplemental Table S2). These results indicate a weak but consistent relationship between the presence of a miRNA-like secondary structure in features and the number of smRNAs that map to those features. We did find some interesting outliers, however. First, the relationship between smRNAs and minMFE statistics was generally not significant for miRNAs (Supplemental Table S2), perhaps reflecting small sample sizes (*n* = 107) or perhaps the fact that miRNA loci generate few distinct smRNAs despite being highly expressed. Similarly, LINE comparisons also were typically not significant; LINEs were heavily saturated for all three smRNA size classes (Supplemental Fig. S6), but few had detectable miRNA-like regions. Second, the estimated linear relationships were typically higher for 21- and 22-nt smRNA than for 24-nt smRNA, which is consistent with their role during the initiation of silencing (Table 3; Supplemental Table S2) and with the observation that *DCL* processing of dsRNA substrates typically yield 21- and 22-nt smRNAs. In genes, for example, correlations between minMFE and 21- to 22-nt smRNAs were again weak but highly significant (R^2^ = 0.01, *P* < 4.12 × 10^−106^), but the correlation with 24-nt smRNAs was not (R^2^ = 8.35 × 10^−05^, *P* = 0.072) (Supplemental Table S2).

**Table 3.**
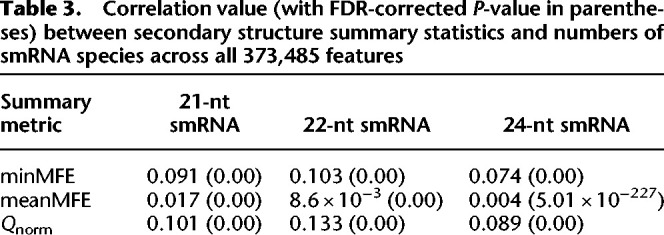
Correlation value (with FDR-corrected *P*-value in parentheses) between secondary structure summary statistics and numbers of smRNA species across all 373,485 features

#### Measuring smRNA abundance with skew

We also examined the relationship between miRNA-like structures and smRNA counts within features by measuring *skew*, that is, the ratio of smRNA mapping in miRNA-like versus non-miRNA-like regions (Methods; Table 1). We defined skew to be zero when smRNA mapping was equivalent on a per nucleotide basis between miRNA-like and non-miRNA-like regions, and skew ranged from −1.0 to 1.0. When it was positive, smRNA mapping was more abundant in miRNA-like regions.

**Figure 3.**
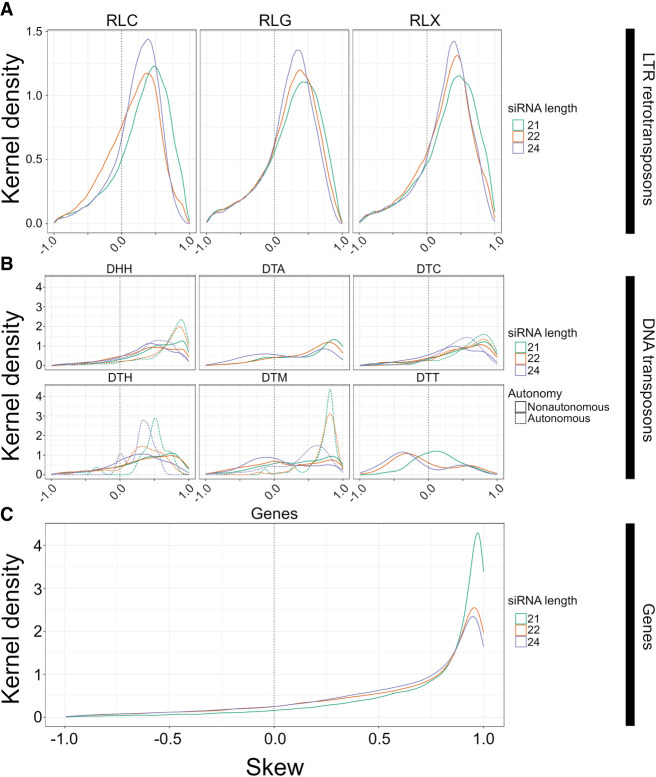
The distribution of skew for smRNA mapping in different feature categories. Skew is presented on the *x*-axis. Height on the *y*-axis represents the Gaussian-estimated kernel density of skew values. Skew measures the relative enrichments of smRNAs in miRNA-like regions compared with non-miRNA regions and ranges from 1.0 (enrichment in miRNA-like regions) to −1.0 (enrichment in non-miRNA-like regions). All panels use the same *x*-axis. The dotted vertical line represents zero, where smRNA density is not skewed to either low- or highMFE regions. (*A*) Skew for retrotransposons for 21-, 22-, and 24-nt smRNAs, separately for *Copia* (RLC), Ty3 (RLG), and unknown retrotransposons (RLX). (*B*) Skew for DNA transposons, with names for the three letter codes provided in Table 2. The dashed lines represent skew for putatively autonomous elements, and solid lines represent nonautonomous elements. (*C*) Skew measured in genes. These graphs are based on LP-hairpins but are analogous for lowMFE regions, and all feature categories are presented in Supplemental Figure S7.

Generally, TEs in all superfamilies showed positive skews, reflecting the tendency for more smRNAs to map to LP-hairpins (Fig. 3A,B) and the lowMFE regions of RF-structured elements (Supplemental Fig. S7). For example, *Copia/RLC* elements had positive skews, with slightly higher skews for 22-nt smRNAs as opposed to 21- and 24-nt smRNAs (Fig. 3A). These results were confirmed by linear mixed-effect models; all three smRNA lengths were significantly higher in *Copia*/RLC LP-hairpin regions with minMFE, meanMFE, and *Q*_*norm*_ (all *P*-values < 1.23 × 10^−04^) (Supplemental Table S2; Supplemental Figs. S8, S9). Overall, LTR elements had more obvious skew than DNA elements, although five of six DNA superfamilies had positive skews for all three smRNA lengths (Fig. 3A), and these observations were largely supported by mixed-effect models (Supplemental Tables S3, S4).

We also examined skew within genes. Genes had homology with far fewer smRNA species than most TE types (nearly 100 times less in most cases) (Supplemental Fig. S6), but smRNA species abundance was roughly equivalent between genes and their transcripts. Although genes mapped fewer smRNAs overall, they had stronger skews than any of the TE superfamilies. For example, roughly threefold more smRNAs (of all size classes) mapped to lowMFE in genes compared with the 1.5- and 1.3-fold difference in *CACTA*/DTC transposons and *Copia*/RLC retrotransposons. This effect was more pronounced for LP-hairpins. Genes had an approximately 89-fold greater smRNA density in LP-hairpins compared with nonhairpin regions, compared with 2.9-fold greater density for LTR retrotransposons (which includes the RLC, RLG, and RLX superfamilies). Linear mixed-effect models were significant for higher smRNA abundance in lowMFE regions and LP-hairpins of genes for all three smRNA lengths (*P* ≅ 0) (Supplemental Tables S3, S4; Supplemental Figs. S8, S9). As a negative control, we analyzed organellar genes because they are typically sequestered from the cytosolic complexes like *DCL* and *RDR6* and hence should not show any skew. smRNAs mapped to organellar genes at low levels but had no skew (Supplemental Fig. S10).

### Expression matters: putatively autonomous vs. nonautonomous TEs

Nonautonomous DNA transposons are not transcribed (except when they are within expressed UTRs or introns), and therefore, RNA secondary structure generally cannot drive the creation of smRNAs for these elements (Panda et al. 2016). We therefore predicted a difference in skew between autonomous and nonautonomous DNA elements. To investigate, we separated DNA transposons into nonautonomous and autonomous elements using transposase homology data (see Methods) (Stitzer et al. 2021) and then repeated our skew and linear model analyses. In most cases, nonautonomous elements had notably less smRNA skew than did autonomous elements (Fig. 3B). This pattern was consistent among *Helitron*/DHH (autonomous mean skew among all smRNA lengths = 0.91, nonautonomous mean = 0.37), *CACTA*/DTC (autonomous mean = 0.44, nonautonomous mean = 0.34), *Harbinger*/DTH elements (autonomous mean = 0.37, nonautonomous mean = 0.27), and *Mutator*/DTM (autonomous mean = 0.51, nonautonomous mean = 0.05), but it was particularly notable for 21- and 22-nt smRNAs (*P* < 7.5 × 10^−31^) among *Helitrons*/DHH and *Mutator*/DTM elements, most of which are nonautonomous in maize (Stitzer et al. 2021). Note that all *Mariner*/DTT elements were nonautonomous, which may relate to their overall lack of skew (Fig. 3B).

### Methylation peaks in miRNA-like regions

**Figure 4.**
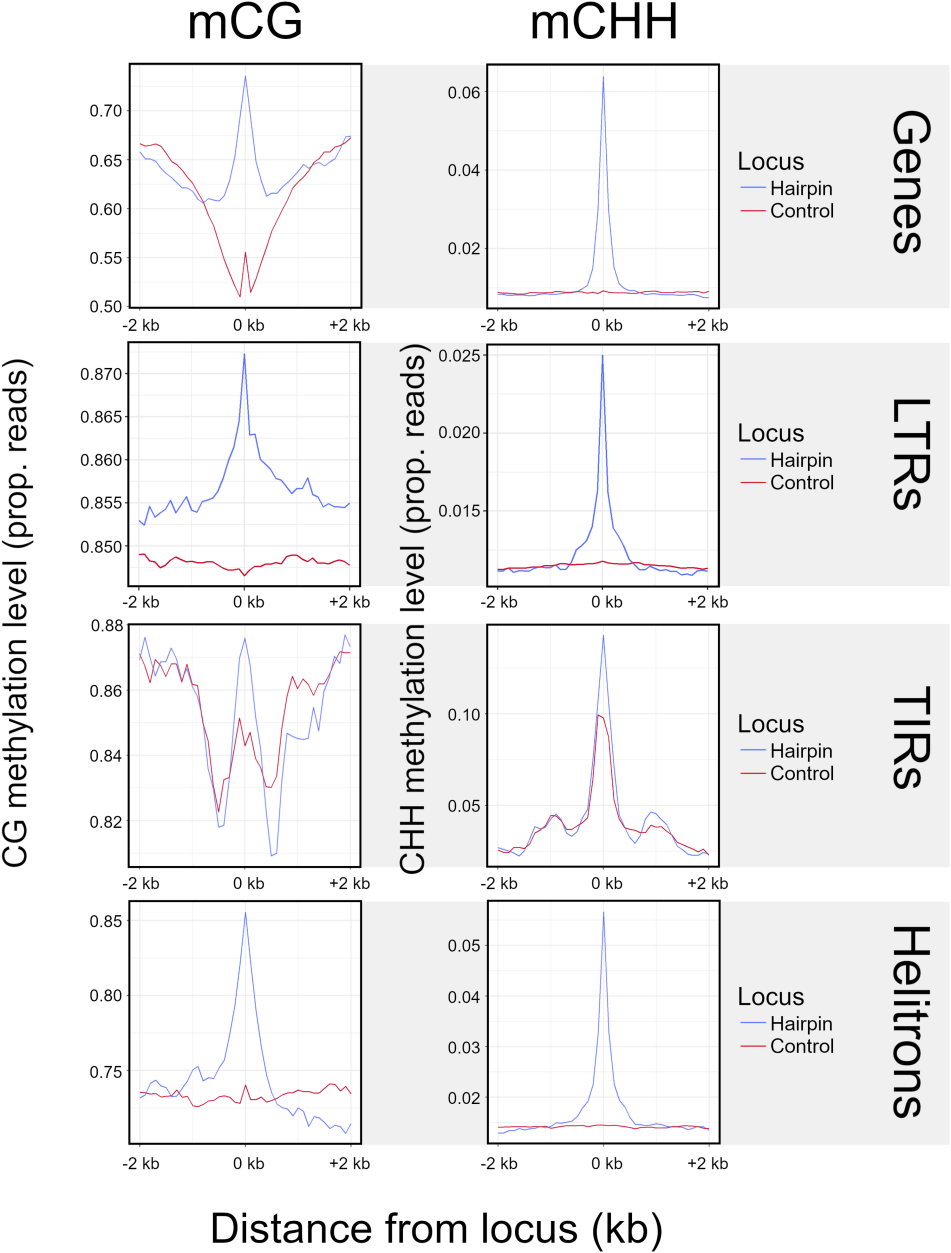
Methylation at LP-hairpins. The *left* column shows methylation in the CG context (mCG), and the *right* column shows methylation in the CHH context (mCHH). Each row represents a different feature type. The blue lines summarize the patterns of methylation in the hairpin (variable sizes, median = 25 nt) across all hairpins in a given feature type (e.g., all TIR hairpins, gene hairpins, etc.) and their flanking regions, divided into 40 nonoverlapping 100-bp windows. We assigned a control window to each hairpin in the data set by choosing a random window of the same size as the hairpin within the same element. The red line corresponds to methylation patterns around these randomized control loci.

One function of smRNAs is to recruit methylases, leading to RNA-directed DNA methylation (RdDM) (Matzke and Mosher 2014). We predicted that miRNA-like structures should be more highly methylated because they map more smRNAs. We further predicted that this effect should be primarily detected in the CHH context, because mCHH is more dependent on RdDM than mCG and mCHG (Law and Jacobsen 2010). We plotted weighted methylation levels (Schultz et al. 2012) from B73 (Hufford et al. 2021), focusing on regions of miRNA-like structure and 2 kb upstream and downstream. Both LP-hairpins (Fig. 4) and lowMFE regions (Supplemental Fig. S11) had peaks of CHH methylation centered on the region; this peak dissipated rapidly, especially for LP-hairpins. These peaks were found in all feature types with detectable miRNA-like structures, including RNA elements, DNA elements, and genes. We also confirmed that miRNA-like regions had significantly higher levels of CHH methylation than other regions by comparing them to randomly chosen unstructured regions of the same length as LP-hairpins (Fig. 4). Finally, we found that CHH methylation levels in LP-hairpins were significantly higher than those in the rest of the corresponding feature sequence (paired *t*-test; *P*-values between 3.43 × 10^−81^ and 1.16 × 10^−165^ among genes, TIRs, LINEs, LTRs, and helitrons), with enrichments as high as ∼10× in genic hairpins.

### miRNA-like structures and gene expression

Genes possess regions with stable RNA secondary structure (Figs. 1, 2), and this secondary structure coincides with the presence of smRNAs (Fig. 3C; Supplemental Tables S3, S4) and methylation (Fig. 4; Supplemental Fig. S11). Yet, genes are usually expressed, which raises the question as to whether these miRNA-like structures have a quantifiable relationship to gene expression. To address this question, we used previously published RNA-seq data from 23 B73 tissues across developmental stages and tissues (Walley et al. 2016). We focused these analyses on structured genes with lowMFE regions (as opposed to LP-hairpins), both because they were common in the UTRs and gene bodies of genes (Fig. 2) and because 5′ secondary structure is known to be important to gene function. In contrast, LP-hairpins were detected in only ∼5% of genic transcripts (Table 2); however, the results presented below for lowMFE regions were often recapitulated with LP-hairpin data.

**Figure 5.**
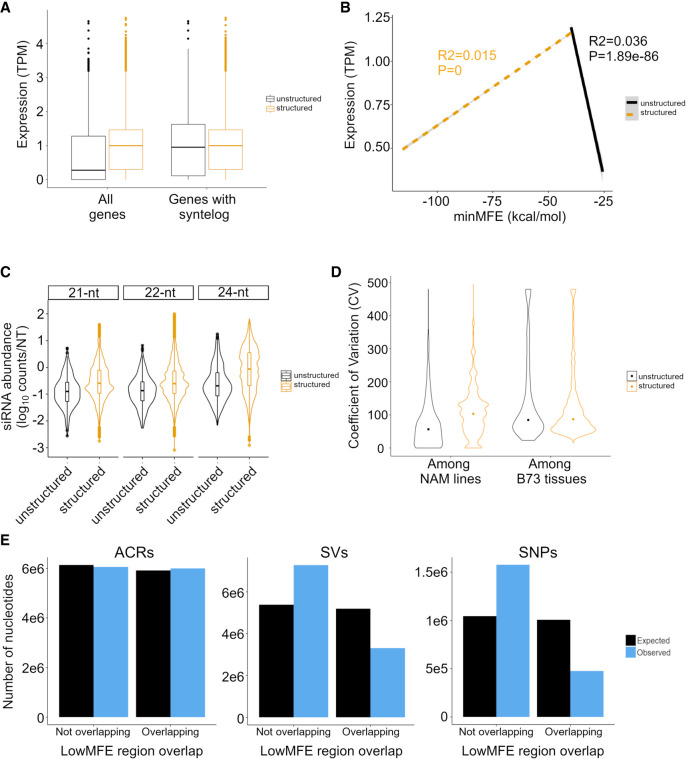
Expression differences between structured and unstructured genes, as defined by RNAfold analysis, in B73. The expression data are based on combined data across 23 tissues. (*A*) Difference in the overall magnitude of expression in all structured (n = 27,034) versus unstructured (n = 5054) genes and in structured versus unstructured genes with a syntelog in *S. bicolor*. The box plots report the range of the middle quartiles; whiskers report the range; and lines represent the median. (*B*) Expression as a function of minMFE for structured (dashed line) and unstructured genes with a *S. bicolor* syntelog (solid line). Both lines report the linear regression; both slopes are highly significant, as indicated by *P*-values on the figure. (*C*) The coefficient of variation (CV) of gene expression across the 26 NAM parents compared between structured and unstructured genes with a *S. bicolor* syntelog. The two categories differ significantly (*P* < 2.22 × 10^−16^). The graph also reports CV among B73 tissues, which does not differ significantly between structured and unstructured genes (*P* = 0.32). (*D*) smRNA mapping to structured and unstructured genes and for three smRNA lengths. For all three lengths, the difference is significant (*P* < 2.22 × 10^−16^). The violin plots show the distributions of smRNA counts, and the boxplots are formatted the same as in *A*. (*E*) Epigenetic and genetic features in lowMFE regions of genes. The plots show the number of expected and observed features overlapping (or not overlapping) the lowMFE region. For example, the number of ACRs (*left* graph) overlapping lowMFE regions is very similar to the number expected, based on the distributions along genes. In contrast, the numbers of observed SVs (*middle*) and SNPs (*right*) are highly underrepresented in lowMFE regions.

We began by comparing expression in 27,025 structured versus 5060 unstructured genes. Structured genes had significantly higher expression (*t*-test, *P* < 2.0 × 10^−16^) (Fig. 5A), and this was true for all tissues (Supplemental Fig. S12), as well as for genes that contained LP-hairpins (Supplemental Fig. S13). We suspected, however, that most unstructured genes either were pseudogenes or were misannotated. To focus on evolutionarily conserved (and hence presumably bona fide) genes, we identified 24,784 B73 genes with syntelogs in *Sorghum bicolor* (see Methods) (Muyle et al. 2021). Among the syntelog set, 16,171 were structured, and 460 were unstructured. Structured syntelogs still had a mean expression level that was slightly higher than unstructured syntelogs (*P* = 3.7 × 10^−4^) (Fig. 5A). More important, however, was the quantifiable relationship between the minMFE and gene expression. Among structured syntelogs, the relationship was significantly positive such that gene expression peaked at a minMFE of ∼40 kcal/mol (Fig. 5B). The opposite was true among unstructured genes because higher expression occurred with lower MFEs (Fig. 5B). This pattern implies the existence of an optimal minMFE for gene expression. These trends are present for many of the 23 separate B73 tissues separately (Supplemental Fig. S14) and for the complete gene set, that is, not just syntelogs (Supplemental Fig. S15).

Among syntelogs, structured genes also mapped significantly more smRNAs than do unstructured genes (Fig. 5C), which raises an interesting question: Could this phenomenon modulate the expression of genes? To examine this idea, we analyzed expression data across the 26 NAM founder lines (McMullen et al. 2009). For these analyses, we assumed that the secondary structure designations predicted in B73 applied to its syntelog across all 26 NAM parents (Hufford et al. 2021). We then compared gene expression among lines using the coefficient of variation (CV) based on expression values that were normalized across eight tissues in each line (see Methods) (Hufford et al. 2021). Our analyses revealed that structured genes had significantly higher CVs than nonstructured genes (*P* < 0.01, permutation test) (Fig. 5D). This was true both for comparisons between all genes in each group and between a down-sampled subset of structured genes that was equal in size to the set of unstructured genes. One concern about this analysis is that the CV is standardized by the mean, which could bias results, but this did not drive our results for two reasons. First, we fitted a linear model of expression CV as a function of B73 gene expression, but the correlation was negative (i.e., more highly expressed genes were slightly less variable across lines; R^2^ = 6.1 × 10^−4^; *P* = 1.5 × 10^−7^, estimate = −0.01). Second, we examined CV across 23 B73 tissues. There was no difference in CV between the structured and unstructured syntelogs across tissues (Fig. 5C), illustrating that the CV metric alone does not explain the significant difference across genotypes.

Can the variable expression of structured genes be explained by smRNAs? We predicted that more smRNAs should lead to more expression variation across lines. To investigate this possibility, we fit a linear model of expression CV as a function of smRNA density and found that CV was positively correlated with smRNA abundance (*P* = 6.7 × 10^−283^; R^2^ = 0.010). To see whether an effect was discernible between structured genes of variable minMFE values (as suggested by Fig. 4B), we separated structured genes into four quartiles based on their minMFE and then plotted the numbers of smRNAs that map to each gene in B73. Consistent with our hypothesis, genes in the lowest minMFE quartile mapped more smRNAs than the other three quartiles for all three smRNA lengths, and minMFE was significantly but weakly correlated with CV in a linear model (*P* = 5.8 × 10^−79^; R^2^ = 0.0031).

This evidence shows that higher CVs for expression are related to the number of smRNAs that map to a gene, but additional factors likely cause (or contribute to) expression variability across NAM genotypes. One potential factor is chromatin accessibility. We assessed whether accessibility varies more in lowMFE genic regions by using ATAC-seq data (Hufford et al. 2021), which defines accessible chromatin regions (ACRs) among NAM parents (see Methods). For each parent, we identified whether ACRs overlapped with lowMFE regions more than unstructured (MFE > −40 kcal/mol) genic regions. We found no difference between the two categories (Fig. 5E). Genetic effects, like SNPs and structural variants (SVs), contribute to gene expression variation across the NAM lines, particularly given that regions of structure can have altered mutation rates (Hoede et al. 2006; Monroe et al. 2022). We therefore also examined SNPs and SVs in these regions, based on the data of Hufford et al. (2021). We found that lowMFE regions were less likely to contain SNPs or SVs than were unstructured genic regions (Fig. 5E).

## Discussion

We have profiled miRNA-like secondary structure in annotated features of the maize genome. To our knowledge, this study is the first to comprehensively catalog such structures, and we have done so by applying two bioinformatic prediction methods. The methods rely on different algorithms (RNAfold vs. LinearPartition), different approaches (overlapping windows vs. no windows), and different characteristics to define miRNA-like regions. By design, the LinearPartition analyses relied on a narrower definition (Fig. 2), and so, there were fewer observations. Yet, the two methods provide largely concurrent insights about miRNA-like regions, including their relative abundances among TE superfamilies (Table 2), their locational biases in some TE superfamilies (Fig. 2), their association with elevated smRNA counts in TEs and genes (Fig. 3), and their genome-wide correspondence to peaks of methylation (Fig. 4).

### Detecting miRNA-like secondary structures

For detecting secondary structure, we have included two positive controls: miRNA precursor loci (Wang et al. 2009) and *Copia*/RLC elements (Bousios et al. 2016). As expected, these two feature categories have extreme statistics based on, for example, the proportion of RF-structured elements (Table 2), the proportion of features with LP-hairpins, and average minMFE (Fig. 1). However, these positive controls also indicate an appreciable false negative rate, because 29% (RF-structure) and 38% (LP-hairpin) of pre-miRNA loci do not have detectable miRNA-like structures.

The methods have additional limitations. We need to first reiterate that the approach was not designed to identify *all* secondary structures. Our goal was to identify regions similar to miRNA precursors, because they are thought to be involved in forming dsRNA substrates that lead to the production of smRNAs. Second, there are limitations to the TE annotation sets. For example, MITEs are not included in either annotation set. MITEs are short nonautonomous elements that are characterized by their tendency to form stem-loop structures and to insert near genes (Bureau and Wessler 1992, 1994), where they are often incorporated in read-through transcripts. They are an interesting topic for additional work, but we can provide no insights about them here. Third, we know that some summaries are biased; for example, minMFE is correlated with feature length, and lowMFE regions are more likely in sequences with high G:C composition. We have addressed these biases by using multiple summary statistics, by randomizing the primary sequence to test for significant evidence of structure, and by using two prediction methods. Finally, we recognize that bioinformatic predictions are approximations that may not correspond to in vivo assessments (Ding et al. 2014).

Nonetheless, despite these limitations, the two distinct prediction methods yield several similar trends, including higher smRNA mapping and methylation levels in miRNA-like regions (Table 2; Figs. 1, 2). One prosaic explanation for these results is that they are caused by systematic biases in the prediction methods, but this seems highly unlikely because (1) error in secondary structure prediction should lead to randomness, namely, inconsistent correlations; (2) the inclusion of false negatives among unstructured elements makes the measured correlations inherently conservative; and (3) the results, although not identical, are largely consistent between prediction methods. Because both genes and TEs show this relationship, we conclude that the association between miRNA-like structure and smRNA abundance is a general characteristic of the maize epigenome.

### miRNA-like regions, epigenetic signals, and potential mechanisms

Given known pathways of miRNA and smRNA biogenesis (O'Brien et al. 2018; Hung and Slotkin 2021), we believe the most likely explanation for the observed association is that miRNA-like secondary structures lead directly to smRNA production via *DCL* mechanisms. This conclusion is bolstered by the fact that smRNA skew is more pronounced for expressed genomic regions (like genes and putatively autonomous elements) for which this mechanism is expected to be most active (Fig. 3). However, we cannot prove that the structure:smRNA correlations are caused by the formation and processing of dsRNA substrates by *DCL* mechanisms. Arguably, the most straightforward way to do so would be to map smRNA libraries from maize mutants lacking *DCL* function. We found no such libraries but did map the available libraries from maize RdDM mutants: *mediator of paramutation1* (*mop1*) and *required to maintain repression2* (*rmr2*) (Barbour et al. 2012; Gent et al. 2014). These mutants affect the repression of TEs that have already been silenced (Barbour et al. 2012); they are thus not particularly good candidates to test the dsRNA-substrate model. We nonetheless assessed the effect of mutants on skew by comparing mutant smRNAs to WT individuals from the same study (Supplemental Fig. S16), but we did not observe any clear or consistent patterns across smRNA lengths or TE superfamilies. These comparisons relied on single libraries and are thus more subject to sampling variability than our other observations, which were based on joint consideration of dozens of smRNA libraries.

Because we cannot prove that processing of dsRNA substrates is a causal mechanism, it is worth considering alternative explanations. For example, structure:smRNA correlations could reflect abundance rather than production; one way this could occur is if smRNAs generated from miRNA-like regions degrade less quickly. It is hard to imagine how this might happen, but it is known that smRNAs that are loaded onto AGO have biases (Mi et al. 2008), and thus some may be more stable with longer half-lives. Another possibility is that these structures correlate with degradation through other, non-*DCL* pathways. Some studies have attempted to correct for degradation and other effects by focusing only on genomic regions in which the proportion of 21-, 22-, and 24-nt smRNAs exceed an arbitrary threshold compared with smRNAs of all lengths (Lunardon et al. 2020). We did not apply such a threshold here, because this approach necessarily assumes that some 21-, 22-, and 24-nt smRNAs should be ignored as biologically uninformative. We did, however, assess overlaps in genomic positions between the annotated, 21- to 24-nt siRNA producing loci of Lunardon et al. (2020) and our miRNA-like hairpin structures. Relative to random chance, we found a modest but significant enrichment in overlapping locations in genes and in all TE superfamilies except SINEs and LINEs (Supplemental Table S5), which generally lack miRNA-like structures (Table 2). These analyses suggest that a subset of our miRNA-like secondary structures correspond to loci thought to produce 21- to 24-nt siRNAs.

As a negative control, we repeated this exercise with a set of annotated loci that do not produce smRNAs within the canonical 21- to 24-nt length range (Lunardon et al. 2020), revealing lower enrichment across all features compared with 21- to 24-nt producing loci (Supplemental Table S5).

Although we cannot document a definitive mechanism, precedent suggests that processing of dsRNA substrates likely contributes to the genome-wide structure:smRNA relationship. If true, then we can add insights about its effects. First, we can estimate the relative amount of smRNAs that are produced via processing of dsRNA substrates compared with other smRNA-generating mechanisms. Across the entire data set of 373,485 features (Jiao et al. 2017), minMFE explains 10% of the smRNA mapping results for 21-nt smRNAs (Table 3), providing a rough estimate for the proportion of smRNAs produced from dsRNA substrates. This value is larger for some metrics within specific feature categories; for example, *Q*_*norm*_ explained 24% of the 22-nt smRNA mapping variation in genes, and meanMFE explained 21% of the 21-nt variation for *CACTA*/DTC elements (Supplemental Table S2). On average, across feature categories and smRNA lengths, the summary statistics minMFE, meanMFE, and *Q*_*norm*_ explained 8% of mapping variation between miRNA-like regions and non-miRNA-like regions (Supplemental Table S2). These low but highly significant values are consistent with the fact that dsRNAs are only one of several routes to smRNA production (Carthew and Sontheimer 2009).

Second, our data show that miRNA-like regions are associated with peaks of elevated methylation (Fig. 4). Because siRNAs guide DNA methylation mechanisms (Law and Jacobsen 2010), these peaks likely reflect causal relationships among structure, smRNAs, and methylation. It is especially notable that these peaks are elevated for CHH methylation, which is deposited de novo each generation and thus represents active methylation mechanisms (Law and Jacobsen 2010). Methylation in these peaks is also elevated in other contexts, for example, the CG context (Fig. 4), such that the peaks resemble mCHH islands. mCHH islands are short (∼100-bp) regions of elevated methylation typically found both upstream of and downstream from genes. They were first identified in rice as associated with MITEs (Zemach et al. 2010). In maize, mCHH islands are associated with several TE types, are found near roughly half of genes, are enriched near highly expressed genes, and are negatively associated with body-methylated genes (Gent et al. 2013; Li et al. 2015; Martin et al. 2021). It is not yet known whether mCHH islands typically correspond to miRNA-like secondary structures, but it is a fitting topic for future investigations.

### TE superfamilies vary in the number and pattern of miRNA-like regions

Our work was motivated, in part, by a lack of knowledge about the incipient stages of plant host recognition that lead to TE silencing (Bousios and Gaut 2016). Because processing of dsRNA substrates remains the only recognized pathway to de novo smRNA production (Hung and Slotkin 2021), we had hoped that characterizing miRNA-like regions would provide clues into properties of host recognition across specific TE superfamilies. Our work does not inform this mystery, except to show that *most* annotated TEs have some miRNA-like regions and also to provide a snapshot of variation across TE superfamilies.

One cannot help but wonder why miRNA-like regions are common within TEs. If secondary structure can lead to the potential for host recognition through smRNAs, there should be selective pressure to lose structure. We suspect that there is a cost to loss related to function. In Sireviruses (the principal representative of the *Copia*/RLC superfamily), there is evidence that palindromic motifs define the *cis*-regulatory region of the LTR (Grandbastien 2015). In fact, studies of different TE families in different organisms have revealed that *cis*-regulatory regions are often arranged as arrays of complex, sometimes palindromic, repeats (Vernhettes et al. 1998; Araujo et al. 2001; Fablet et al. 2007; Ianc et al. 2014; Martínez et al. 2016), implying that secondary structures often assume a *cis*-regulatory function. We hypothesize that *Copia*/RLC elements are engaged in a tug of war between the functional necessities of secondary structure and the tendency of these same regions to act as templates for smRNAs. We presume similar dynamics apply to other TE superfamilies, although clearly this conjecture requires further detailed analyses of structure and function in specific TEs.

### Genes: evidence for a trade-off

Our analyses have uncovered a few unexpected features of genes. One is that the two methods provide different insights. The RNAfold approach identifies 85% of genes as RF-structured (Table 2), with an evident bias toward 5′ UTR regions (Fig. 2). This result is not unexpected, given that secondary structures in 5′ UTRs are tied to crucial functions in ribosome binding and translation (Babendure et al. 2006; Matoulkova et al. 2012). In contrast, LP-hairpins are primarily found in introns. We conclude that 5′ UTRs commonly have miRNA-like regions (as defined by MFEs) but apparently lack the stem-loop structures identified by LinearPartition. Nonetheless, both lowMFE regions and LP-hairpins associate positively with smRNAs and show elevated CHH methylation levels within genes (Figs. 3, 4; Supplemental Fig. S11).

This is not the first such observation for plant genes, because Li et al. (2012) discovered that *Arabidopsis* mRNA transcripts with more stable secondary structures had higher smRNA expression with lower genic expression. Our work expands this previous work in two ways. First, we have extended the observations to maize; it is notable that genes in maize and *Arabidopsis* share these trends because maize has a larger genome with more TEs. Second, we have shown that secondary structure does not universally correlate negatively with gene expression. Rather, the relationship is tiered: There is a qualitative difference in expression between genes with and without RF-structure (Fig. 4A,B), probably reflecting that secondary structure in 5′ UTRs is crucial for some aspects of gene function. Among genes with RF-structure, however, genes with strong structure (as measured by minMFE) tend to be less expressed than genes with moderate RF-structure (Fig. 5B). That is, genes with particularly strong secondary structures (i.e., very low MFEs) have lower expression.

This relationship suggests that there can be “too much of a good thing” when it comes to miRNA-like structures. The potential functional consequence of “too much” is illustrated across the NAM parental genotypes because structured genes with higher coefficients of variation tend to map more smRNAs (Fig. 5B) and have more variable expression among genotypes (Fig. 5C). We investigated whether this observation could be explained by other features of the miRNA-like regions, such as especially high variability in chromatin accessibility or high numbers of SNPs or SVs, because some work has shown that structured regions can have higher mutation rates (Hoede et al. 2006). However, none of these variables explain higher expression variation across genotypes. In fact, the miRNA-like regions tend to have fewer SNPs and SVs than the rest of the gene (Fig. 5E), suggesting that the miRNA-like regions are under purifying selection.

Altogether, these results suggest the possibility of an evolutionary tradeoff between selection for stable secondary structure against too much secondary structure. Even so, we are still left by a paradox: If genes have miRNA-like regions that serve as a template for smRNA production, why are they not silenced? We do not have the answer, but we believe it must rely on the bevy of differences between heteromatin and euchromatin. It is known, for example, that genic regions have distinct sets of chromatin markers relative to heterochromatin and also that demethylases like *Increased in Bonsai Methylation 1* (*IBM1*) and *repressor of silencing 1* (*ROS1*) (Gong et al. 2002; Penterman et al. 2007) actively demethylate expressed genes (Saze et al. 2008; Miura et al. 2009). Some aspects of genic methylation are under selection (Muyle et al. 2022), and selection will be particularly strong against mechanisms that silence genic regions. These mechanisms may have evolved in part to counter the potentially deleterious effects of the formation of dsRNA structures and subsequent production of smRNAs.

## Methods

### B73 annotation and secondary structure prediction

Version 4 of the B73 maize genome and version 4.39 of the genome annotation were downloaded from Gramene (https://www.gramene.org). B73 TE annotations were retrieved from https://mcstitzer.github.io/maize_TEs/ (Jiao et al. 2017; Stitzer et al. 2021). The data were filtered for redundancy, and then both BED and FASTA files were generated. From each feature, 110-nt sliding windows (with 1-nt step size) were fed into RNAfold v2.4.9 from ViennaRNA (Lorenz et al. 2011). Summary statistics (minMFE, meanMFE, and lowMFE) were calculated for each feature, based on all windows in that feature. To determine whether a feature contained significant structure, the feature sequence was randomized by shuffling nucleotide positions five times across the feature length, calculating minMFE each time. The significance of observed structure versus the five randomizations was calculated using a Wilcoxon one-sided test with Benjamini–Hochberg correction in R (v. 4.1.0) (R Core Team 2022). We plotted lowMFE regions across features (Fig. 2; Supplemental Fig. S4) by splitting each feature into 100 equally sized bins and counting the number of <−40 kcal/mol regions overlapping each bin. Motifs within lowMFE regions were analyzed by the MEME motif finder (v5.4.0) (Bailey and Elkan 1994) using the DNA alphabet in classic mode and selecting the top 10 overrepresented sequences for each category.

We used LinearPartition v1.0 (Zhang et al. 2020) to annotate LP-hairpins. We ran LinearPartition with default arguments on each feature sequence, outputting the partition function, *Q*, and the matrix of base-pairing probabilities. *Q*_*norm*_ was calculated by dividing *Q* by the length of each feature. We used the base-pairing matrix to infer the locations of miRNA-like hairpins by searching for consecutive runs of likely pairing bases in R, using functions from the IRanges and GenomicRanges (Lawrence et al. 2013), data.table (Dowle and Srinivasan 2023), and tidyverse (Wickham et al. 2019) packages. We focused on bases with more than 0.90 pairing probabilities and required LP-hairpins to be ≥21-nt long with fewer than five mismatched nucleotides (fewer than three mismatches in asymmetric bulges) (Axtell and Meyers 2018), without an upper limit on length.

### smRNA library analysis

smRNA-seq libraries were downloaded using NCBI Sequence Read Archive (SRA) tools and SRAExplorer (https://github.com/ewels/sra-explorer) from the sources indicated in Supplemental Table S1. Adapters, regions with low quality, and low-quality reads were trimmed using FastQC and cutadapt v0.39 (Martin 2011). The list of adapters for each library is included in Supplemental Table S6. Trimmed reads were filtered and split based on size, matching 21, 22, and 24 nt in length. We identified unique smRNA sequences, which we refer to as “species,” following previously described methods (Bousios et al. 2016, 2017). smRNA species were mapped to B73 V4 using Bowtie 2 v2.4.2 (Langmead and Salzberg 2012), preserving only perfect alignments. SAMtools v1.10 (Danecek et al. 2021) was used to convert and sort the alignment output. At each nucleotide, both uniquely and nonuniquely mapping smRNAs were used to calculate the number of smRNA species; strand was not taken into account. Normalization was performed when comparing sequence regions of different lengths by summing counts and dividing by region length.

Correlations between smRNA species density and miRNA-like regions were fitted using the base R (v4.1.0) lm() function. To fit these models, smRNA species were summed across all libraries for each feature. These linear models can be expressed as *log(smRNA counts per kilobase across feature + 1)* ∼ *secondary structure metric*.

To test the significance of differences in smRNA species density between high and low MFE regions within features, mixed-effect models were fit for each smRNA size class using the R package *lme4* (Bates et al. 2015). In these models, smRNA mapping counts from each library were not combined, meaning that each smRNA library:feature pair was counted individually. These mixed-effect models can be expressed as *log(smRNA counts per kb across region + 1)* ∼ *structure designation + (1|feature)*.

Skew (Fig. 4) was calculated for each TE superfamily and genes as

$$\frac{hairpin\left(\frac{species}{nt}\right)-nonHP\left(\frac{species}{nt}\right)}{hairpin+nonHP\left(\frac{species}{nt}\right)}.$$

For these calculations, feature-library pairs with zero smRNA species in either miRNA-like or non-miRNA-like regions were removed from each data set. We tested whether skew differed from zero using Wilcoxon one-sided tests in R.

Autonomous versus nonautonomous designations for TEs were defined depending on TE type, but they were determined based on the presence or absence of open reading frames within the TEs, as identified by Stitzer et al. (2021; downloaded from https://github.com/mcstitzer/maize_genomic_ecosystem). TIRs were considered autonomous if they contained sequence homology with a transposase, and helitrons were considered autonomous if they contained *Rep*/*Hel* (Stitzer et al. 2021).

### Methylation analyses

Preprocessed B73 genome-wide methylation data from Hufford et al. (2021) were downloaded from https://datacommons.cyverse.org/browse/iplant/home/shared/NAM/NAM_genome_and_annotation_Jan2021_release/DNA_METHYLATION_UMRs/DNA_methylation_coverage_bigwig_files/NAM_methylation_coverage_on_B73v5_coordinates. We converted V5 coordinates to V4 using the EnsemblPlants CrossMap (v0.6.4) converter. For each region of interest, we calculated the weighted methylation level for each cytosine sequence context (CG or CHH) by dividing the number of methylation-supporting mapped cytosines by the total number of cytosines in the reference within that region (Schultz et al. 2012). To find random control regions to compare to miRNA-like regions, we randomly assigned a region of equal size to the miRNA-like region in the feature that did not overlap with it. We did not consider features for which over half of the features fell within miRNA-like regions, because random control regions could not be determined.

### B73 RNA-seq analyses

B73 gene expression data were downloaded from the ATLAS expression database (www.ebi.ac.uk/gxa/) in transcripts per million (TPM) based on RNA-seq data from 23 maize tissues (E-GEOD-50191) (Walley et al. 2016). The statistical significance of differences between expression of genes in different structure classifications was determined using unpaired *t*-tests, implemented with t.test() in R. Linear models of expression versus secondary structure were separately fit for expression in each tissue type with lm() in R and graphed using ggplot2 (Wickham 2016). These linear models can be expressed as *log(gene expression + 1)* ∼ *MFE metric.* For analysis of syntelogs, we focused on genes with *S. bicolor* syntelogs listed in Supplemental Table S10 of Muyle et al. (2021).

### Comparative analyses among NAM founders

For comparisons across NAM lines, we analyzed data from genes that were shared among all lines (as determined by Hufford et al. 2021). Expression, ATAC-seq, SNP, and SV data for each NAM line were downloaded with B73 coordinates from CyVerse at https://datacommons.cyverse.org/browse/iplant/home/shared/NAM/NAM_genome_and_annotation_Jan2021_release. Gene IDs were converted to V4 using the EnsemblPlants ID history converter (https://plants.ensembl.org/Zea_mays/Tools/IDMapper). Coordinates of TEs and structured regions were converted using the EnsemblPlants CrossMap (v0.6.4) converter with the B73_RefGen_v4 to Zm-B73-REFERENCE-NAM-5.0 parameter. Normalized expression data were downloaded in RPKM format from merged RNA-seq libraries from https://datacommons.cyverse.org/browse/iplant/home/shared/NAM/NAM_genome_and_annotation_Jan2021_release/SUPPLEMENTAL_DATA/pangene-files. The data included RNA-seq normalized across eight tissues in each line: primary root and coleoptile at 6 d after planting, base of the 10th leaf, middle of the 10th leaf, tip of the 10th leaf at the vegetative 11 growth stage, meiotic tassel and immature ear at the V18 growth stage, and anthers at the reproductive 1 growth stage.

The CV of expression was calculated for each gene among the 26 lines using normalized RPKM expression data from Hufford et al. (2021). We calculated CV using the sd() and mean() functions in base R. We determined statistical significance of differences between categories using unpaired *t*-tests in R. We also built a linear model with lm() in R to correlate the magnitude of gene expression in B73 with the CV of that gene across lines: *log(B73 expression + 1)* ∼ *NAM line CV*.

We also measured epigenetic and genetic features across the NAM lines and tracked their overlap with miRNA-like regions. For the former, we concatenated ACRs that overlapped positions between lines, producing a set of merged ACRs. We produced these merged sets using the R libraries IRanges and GenomicRanges (Lawrence et al. 2013). We also extracted the positions of SNPs from the filtered VCF file from Hufford et al. (2021). The expected overlap was calculated as the proportion of genic space taken up by low MFE regions × the total length of features. We assessed overlap between ACRs/SVs/SNPs and miRNA-like regions using GenomicRanges in R.

## Data access

Custom scripts for these analyses are available as Supplemental Code and at GitHub (https://github.com/GautLab/maize_te_structure). Additional Supplemental Files are also available as Supplemental Material and at Figshare (https://figshare.com/projects/siRNAs_and_secondary_structure_in_maize_genes_and_TEs/150714).

## Supplementary Material

Supplement 1

Supplement 2

Supplement 3

Supplement 4

Supplement 5

Supplement 6

Supplement 7

Supplement 8

Supplement 9
