# Supplementary material for: miRNA-like secondary structures in maize (Zea mays) genes and transposable elements correlate with small RNAs, methylation, and expression
Source: Genome Res. 2023 Nov;33(11):1932–46. doi: 10.1101/gr.277459.122 (PMC10760457; doi:10.1101/gr.277459.122)
Supplement: Supplement 2 [file Supplemental_Figures.pdf]

Figure S1

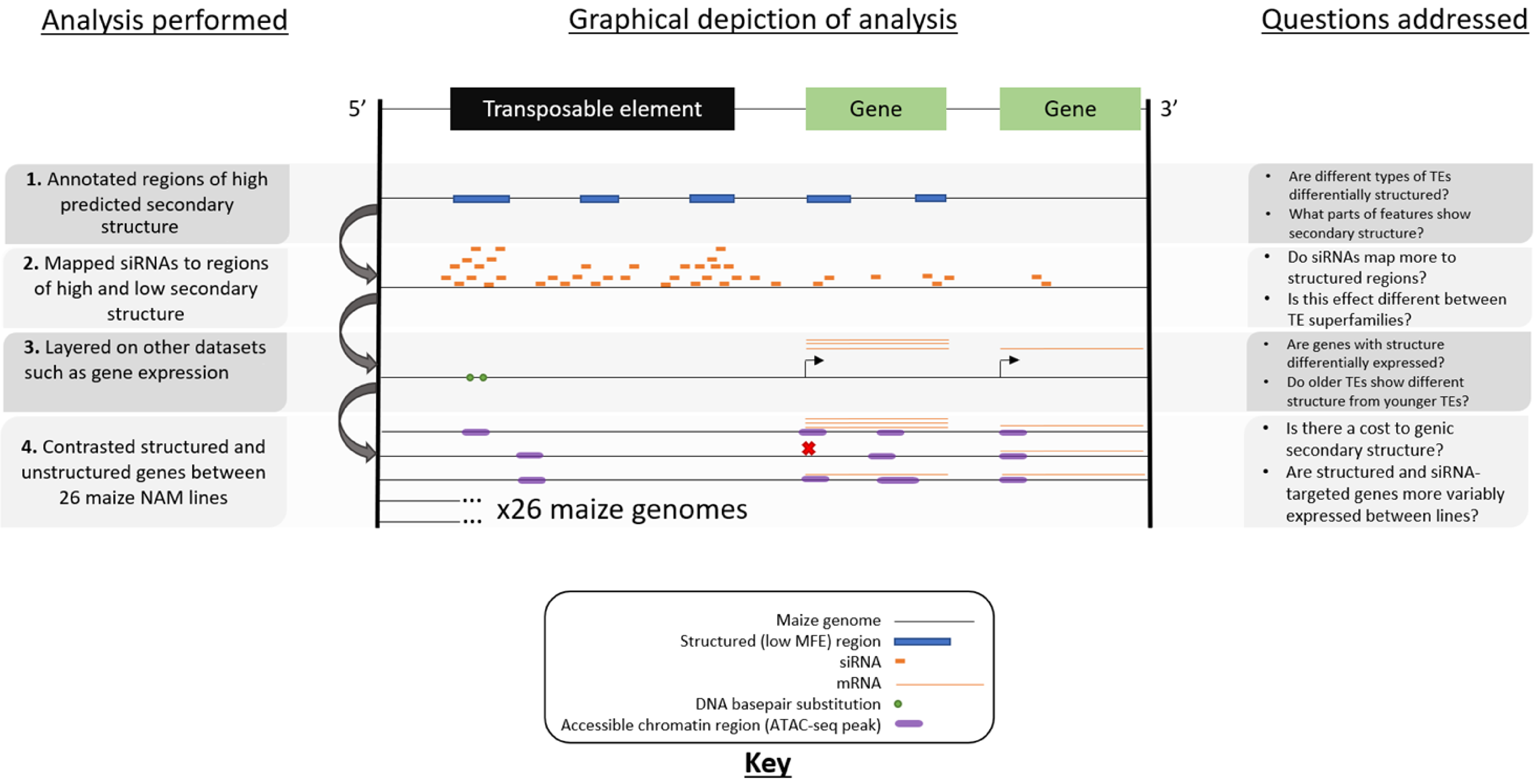

Figure S1: Scheme of analyses carried out. Each sequential layer includes an additional analysis performed on annotated features, including genes, ncRNA loci, and TEs. We (1.) annotated regions of predicted miRNA-like secondary structure (Figs 2-3; Table 1), then (2.) mapped siRNAs across features of varying secondary structure and between regions with miRNA-like structure and without (Fig 4; Table 2), then (3.) compared expression (Fig 5) , and (4.) examined underlying genetic and epigenetic features of genes with differing secondary structure between 26 inbred NAM lines representing the breadth of global maize diversity (Fig 6)

Figure S2

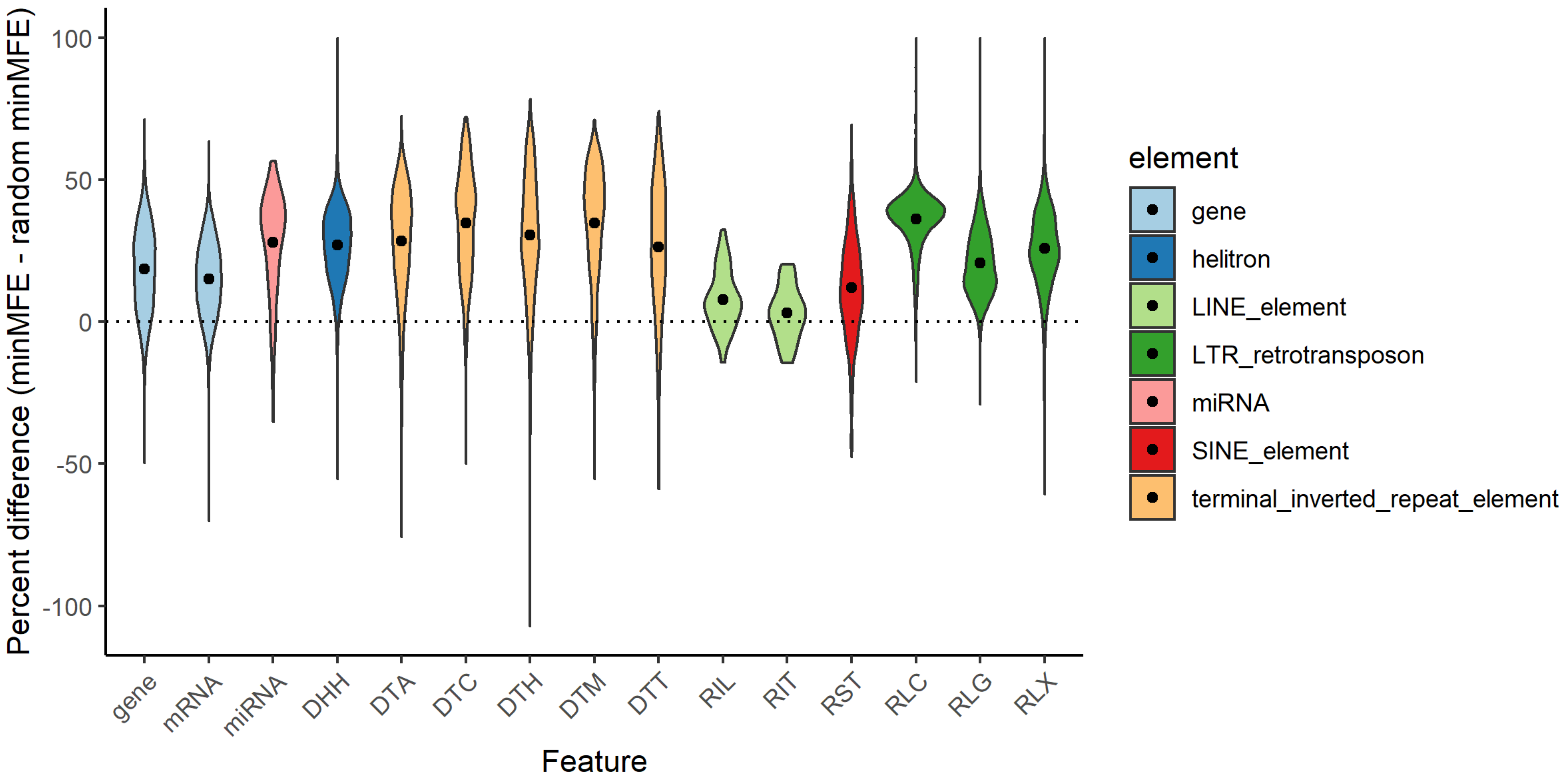

Figure S2. Distributions of percent differences between observed and random minMFEs in each feature type. Differences represent how much *more negative* (and therefore more stabily structured) observed minMFEs were compared to mean minMFE across five randomizations. To find percent differences, these differences were divided by the observed minMFE and multiplied by 100 [e.g., if the observed minMFE was -100 and the mean randomized minMFE was -50, percent difference would be ((-100 + -50) / -100) * 100 = 100%]. Superfamilies are colored by their broader TE category (LTR, TIR, etc.) and dots represent the mean of each distribution. The dotted line represents 0%, or zero difference from random minMFE.

Figure S3

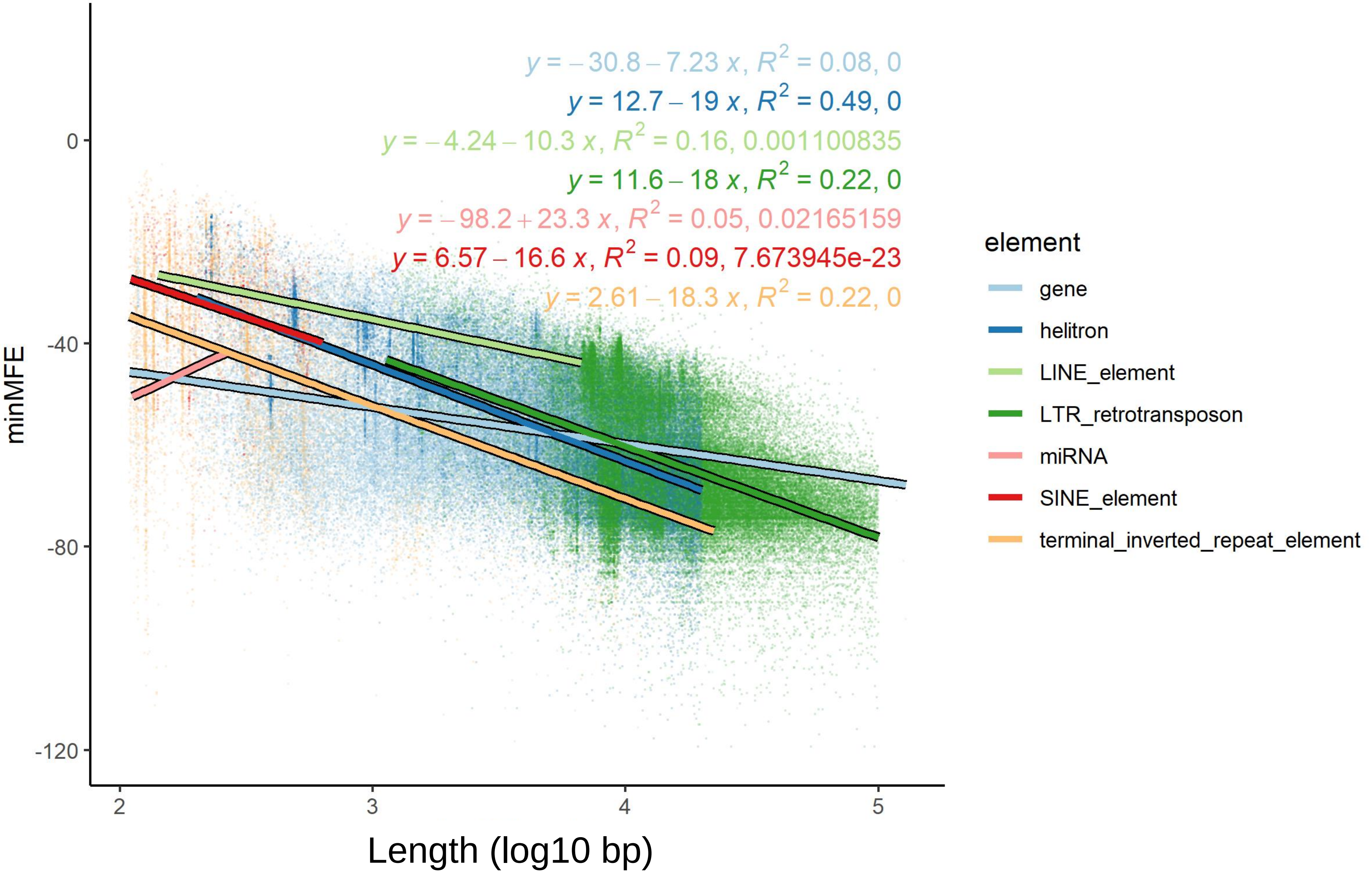

Figure S3: Linear models of minMFE as a function of length. Each type of TE was modeled separately using its length from 5' to 3' end and observed minMFE value. Plots represent simple linear models from the lm() function in R, and colored text represents the formulae, R^2 value, and p-value of each regression.

Figure S4

A

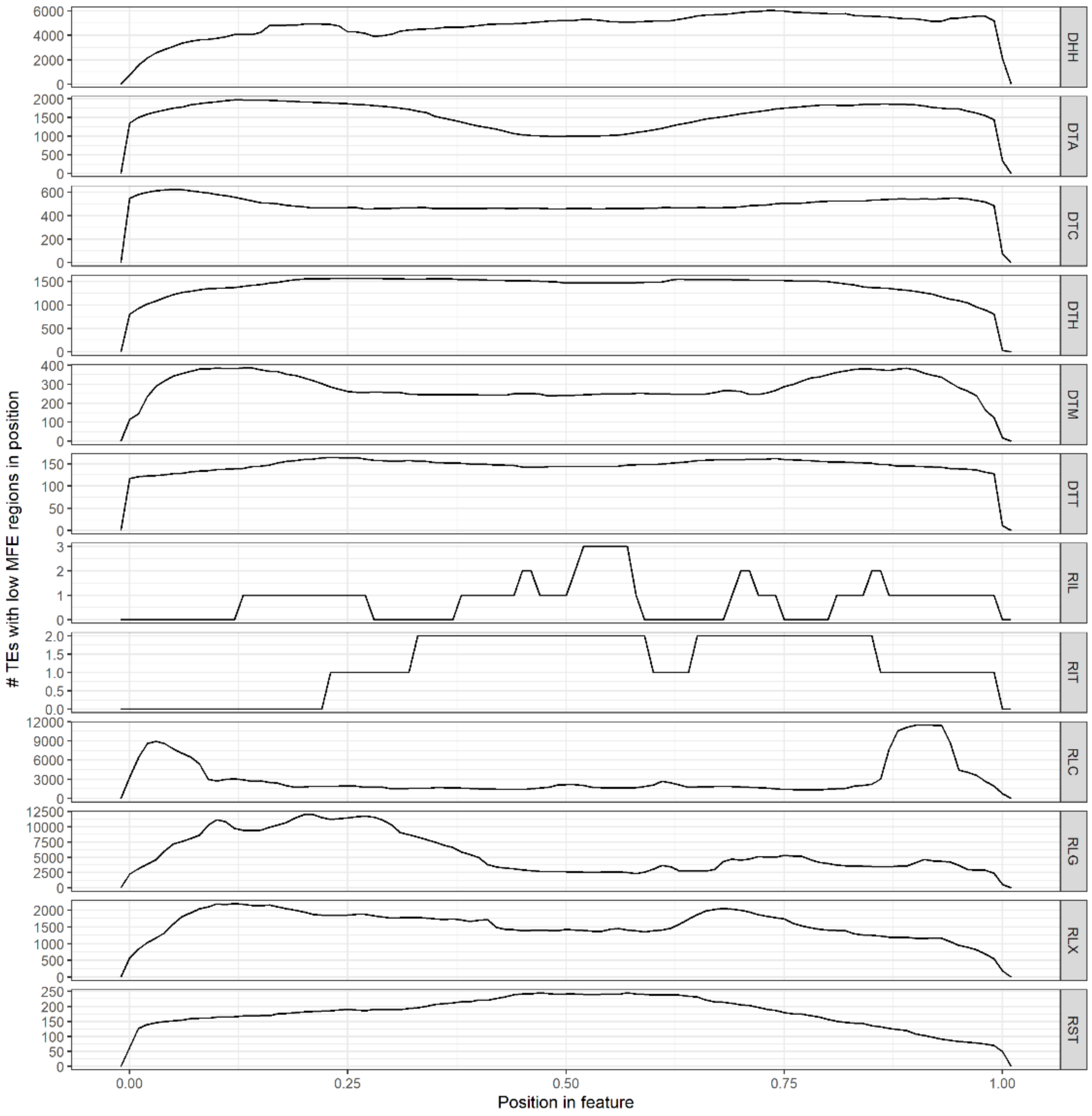

Figure S4. Landscapes of miRNA-like regions across feature types. (**A**) Metaprofiles of lowMFE regions across TE superfamilies, and (**C**) metaprofiles of LP-hairpins across TEs. Each row represents a metaprofile combining data from all members of each feature type. Features were divided into 100 equally sized bins from the 5' end to the 3' end, and the number of features with miRNA-like regions overlapping each of these bins was counted. A peak in the landscape therefore represents a region of the feature type which often shows very stable secondary structure. All rows share the same X axis, which is represented proportionally across the length of the feature from 0.00 (5' end) to 1.00 (3' end).

Figure S4 (cont.)

B

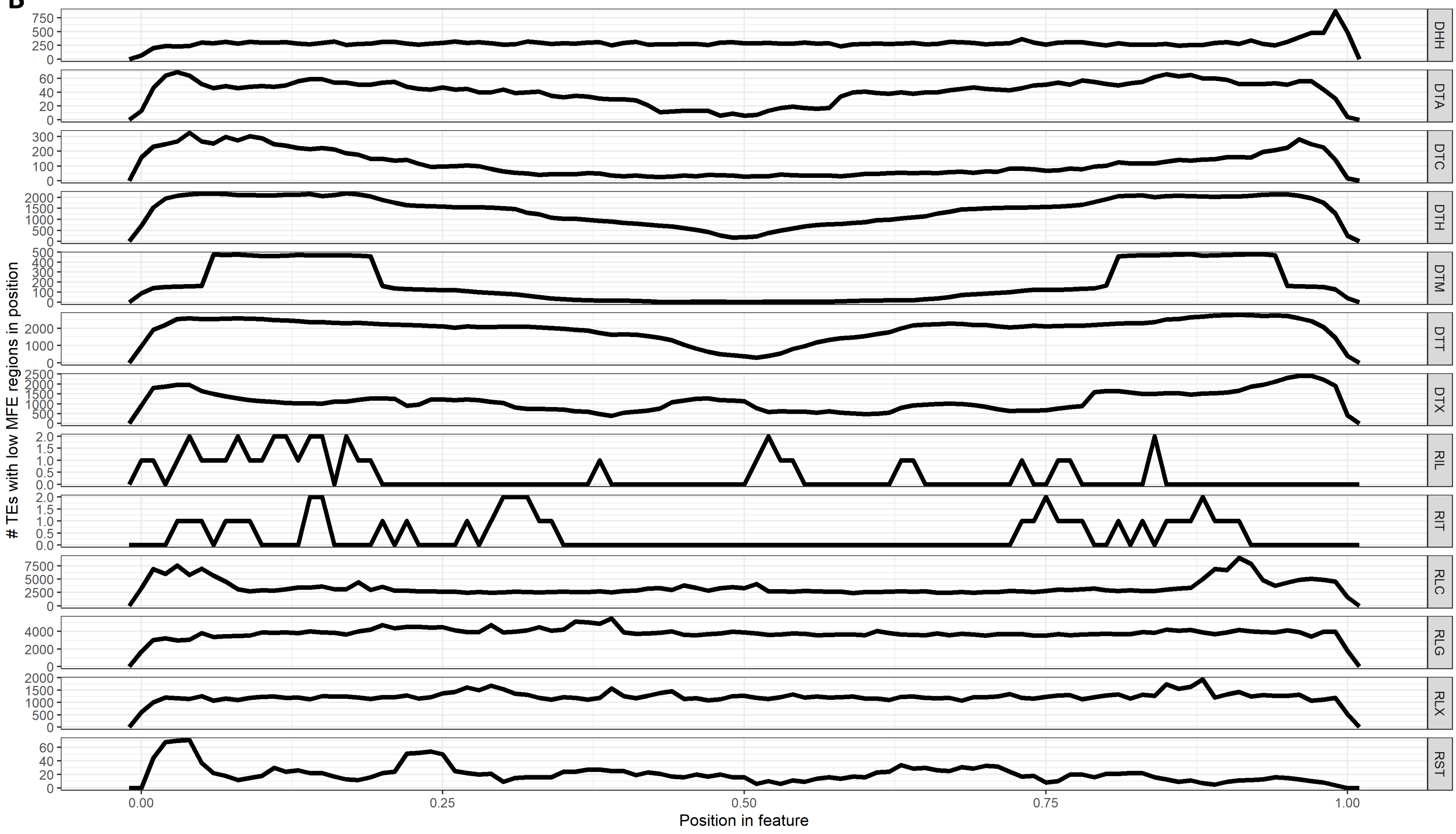

Figure S4. Landscapes of miRNA-like regions across feature types. (**A**) Metaprofiles of lowMFE regions across TE superfamilies, and (**C**) metaprofiles of LP-hairpins across TEs. Each row represents a metaprofile combining data from all members of each feature type. Features were divided into 100 equally sized bins from the 5' end to the 3' end, and the number of features with miRNA-like regions overlapping each of these bins was counted. A peak in the landscape therefore represents a region of the feature type which often shows very stable secondary structure. All rows share the same X axis, which is represented proportionally across the length of the feature from 0.00 (5' end) to 1.00 (3' end).

Figure S5

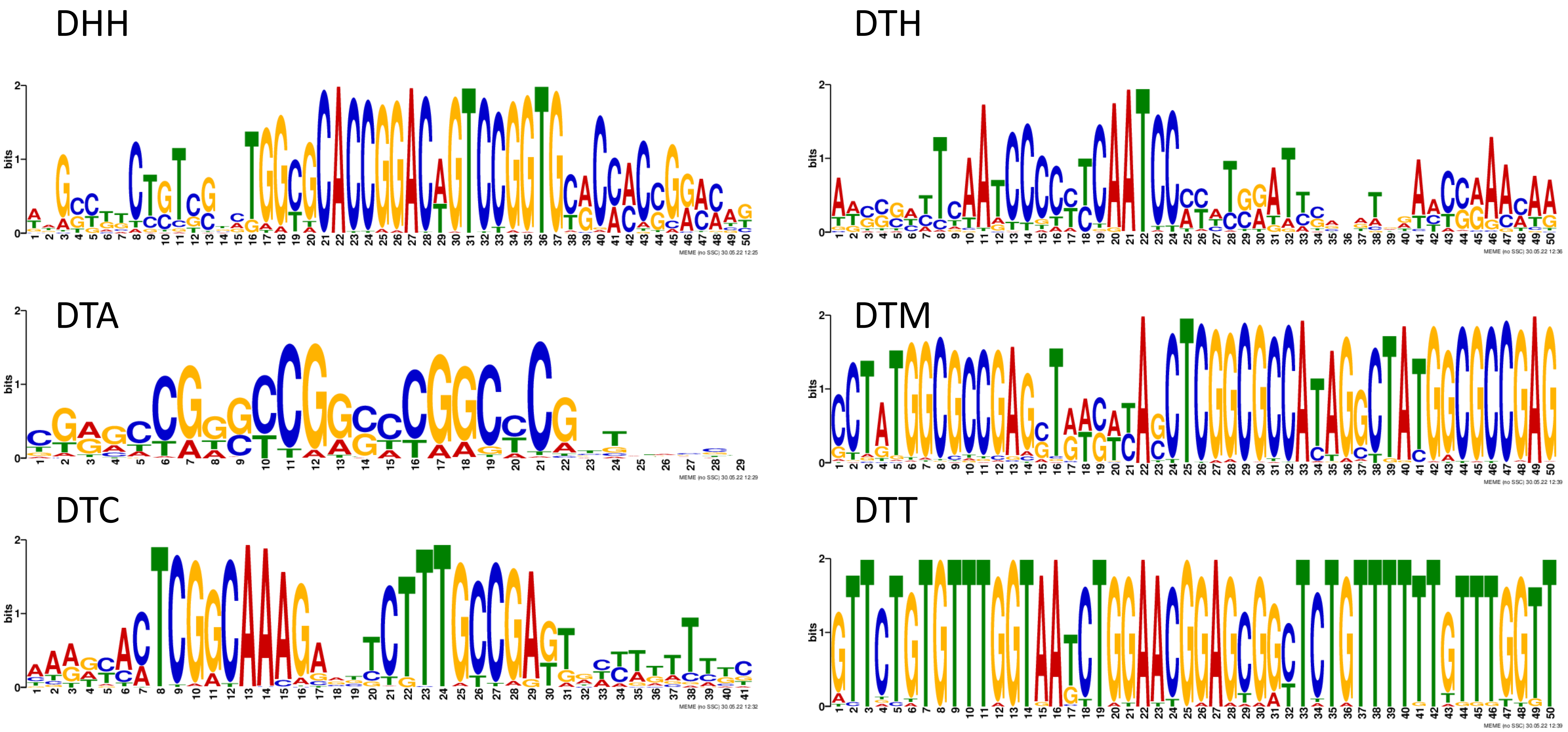

Figure S5. Overrepresented motifs in structured/low MFE regions (<-40 kcal/mol) of structured features. Structured regions of each superfamily were entered into MEME motif finder (See Methods), and logos represent the most highly overrespresented motif found in each superfamily.

Figure S5 (cont.)

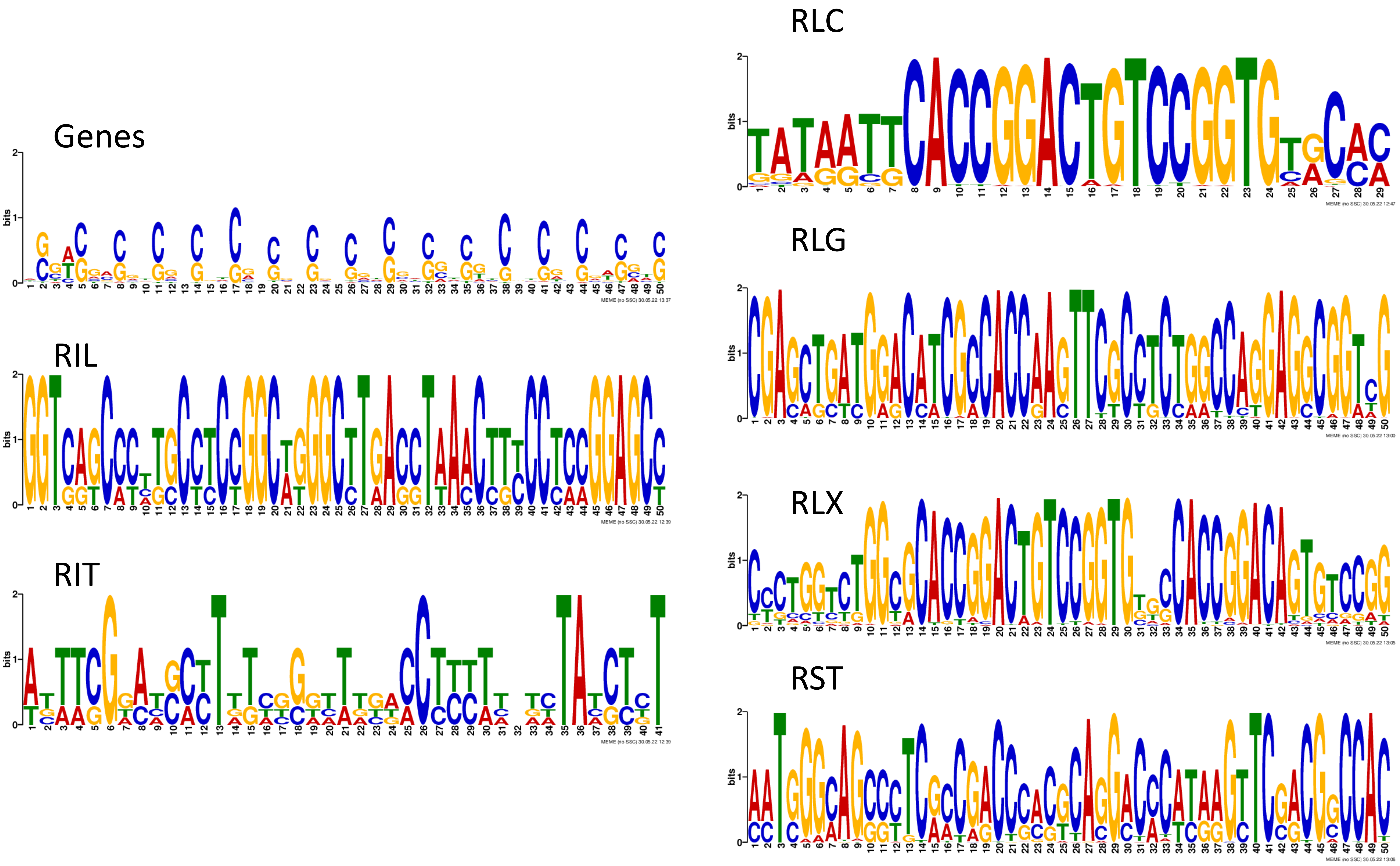

Figure S5. Overrepresented motifs in structured/low MFE regions (<-40 kcal/mol) of structured features. Structured regions of each superfamily were entered into MEME motif finder (See Methods), and logos represent the most highly overrespresented motif found in each superfamily.

Figure S6

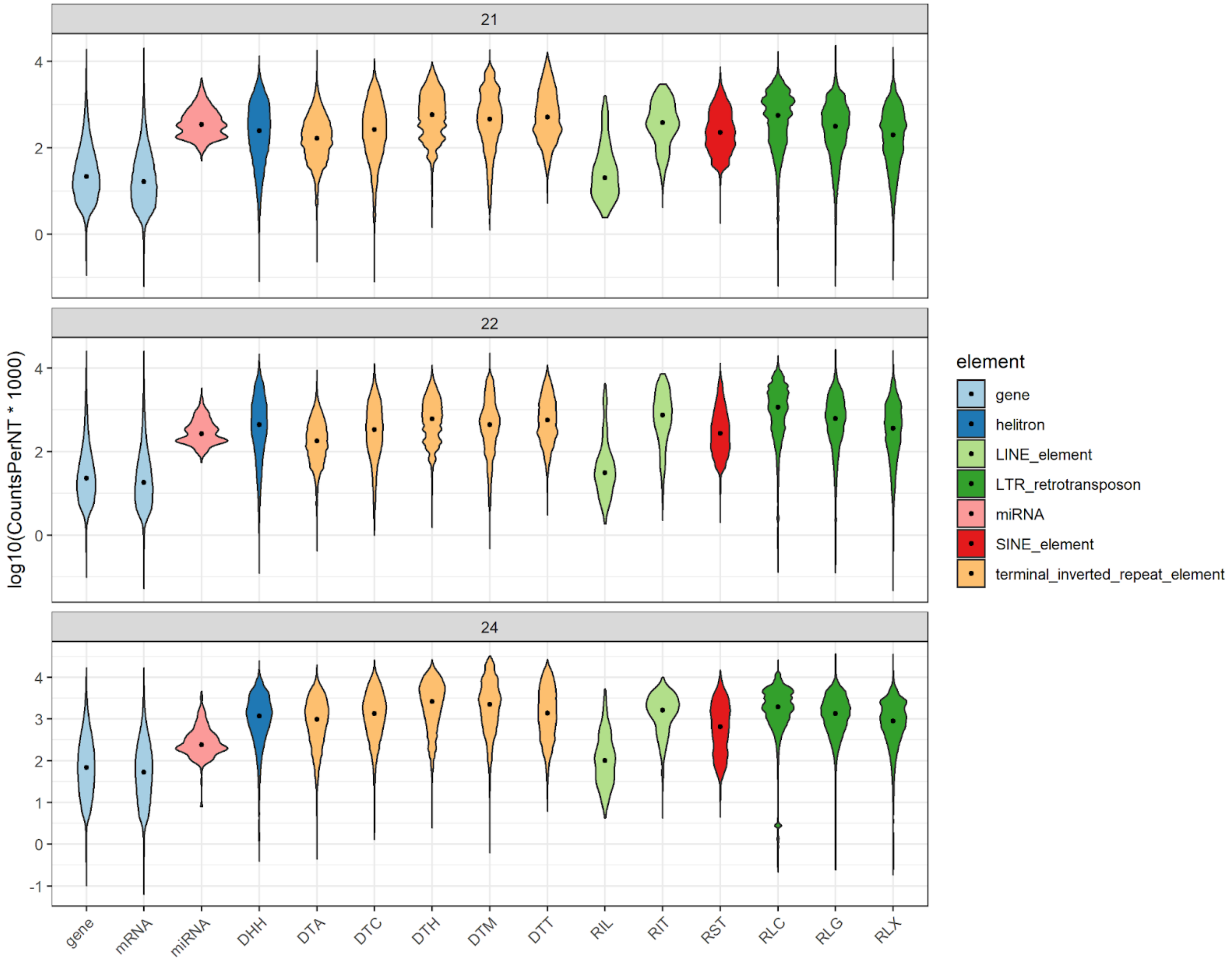

Figure S6. Variation in siRNA mapping between feature types. Violin plots show the distributions of siRNA mapping densities in log10(siRNA species counts per kilobase) for each superfamily/genomic feature, and black dots show the mean of the distribution. Panels represent siRNA size classes (21-nt, 22-nt, 24-nt).

Figure S7

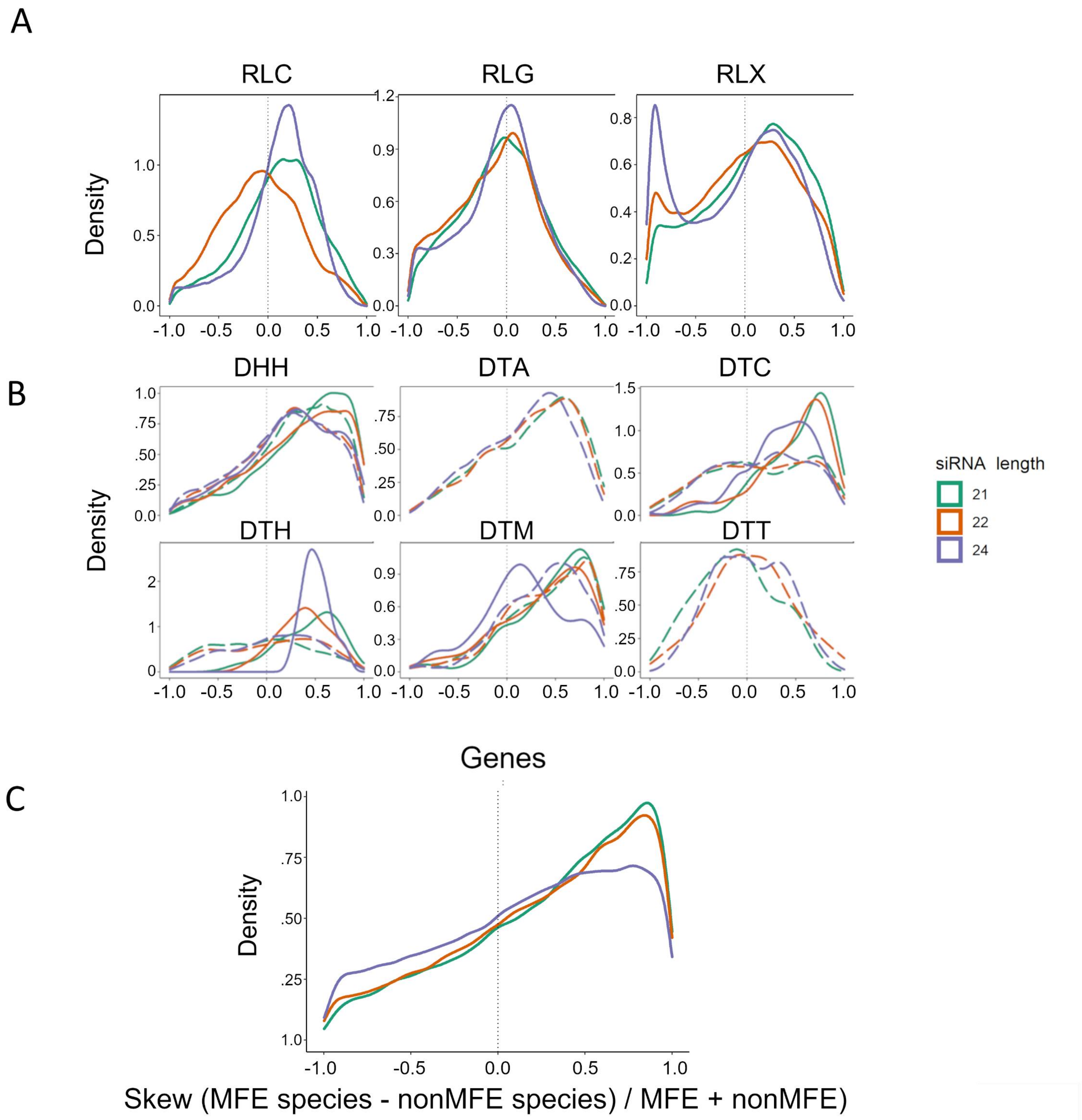

Figure S7. siRNA mapping skew towards lowMFE regions. All panels use the same x-axis, which is a measure of skew, and the dotted vertical line represents zero where smRNA density is not skewed to either low or high MFE regions. A. Retrotransposons and their skew for 21, 22 and 24-nt siRNAs, representing Copia (RLC), Ty3 (RLG) and unknown retrotransposons (RLX). B. DNA transposons, with names for the three letter codes provided in Table 2. The solid lines represent autonomous elements, while dashed lines represent non-autonomous elements. C. Skew measured in genes.

Figure S8

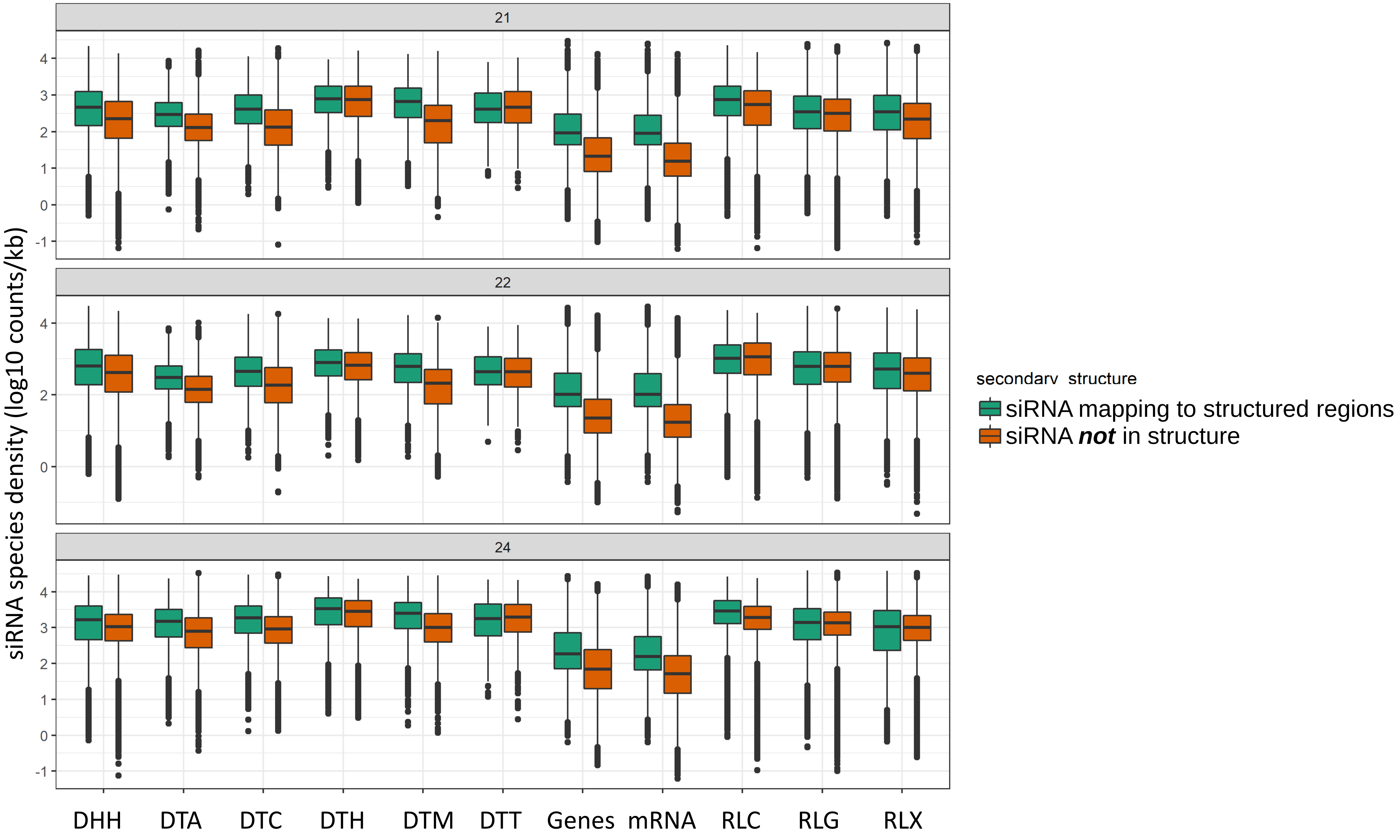

Figure S8. siRNA species mapping density in lowMFE regions vs unstructured regions. For each structured feature (minMFE significantly lower than mean randomized minMFE (see **Methods**), siRNAs mapping to the feature were divided into those mapping to lowMFE regions (<-40 kcal/mol) and those outside of structured regions. Boxplot central lines show the median, and boxes show the 25% and 75% quartiles. Statistical significance in these comparisons can be seen in **Table S3**.

Figure S9

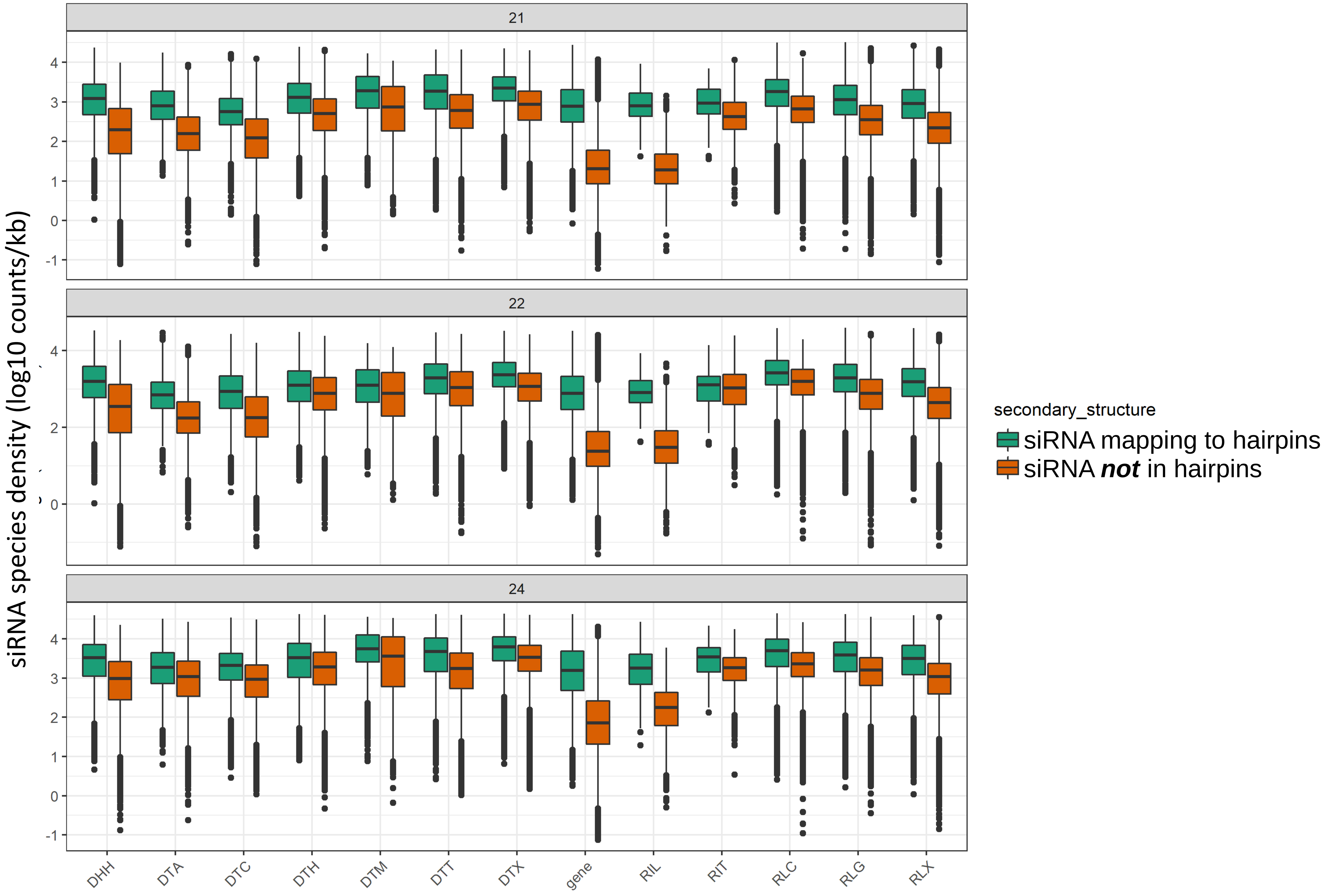

Figure S9. siRNA species mapping density in LP-hairpins vs other regions. For each feature, siRNAs mapping to the feature were divided into those mapping to LP-hairpins (<-40 kcal/mol) and those outside of structured regions. Boxplot central lines show the median, and boxes show the 25% and 75% quartiles. Statistical significance in these comparisons can be seen in **Table S4**.

Figure S10

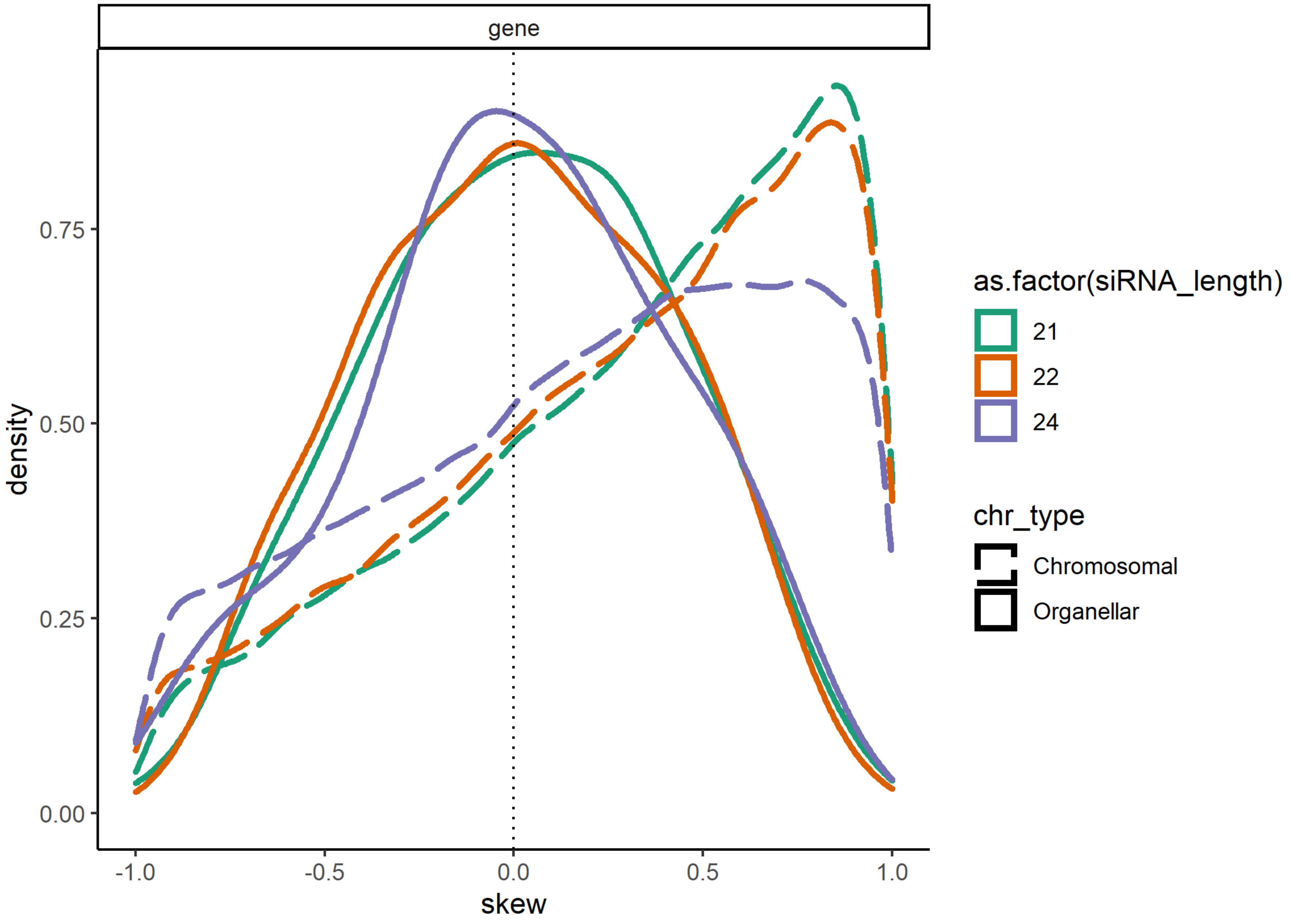

Figure S10. siRNA mapping skew towards lowMFE regions in organellar vs nuclear genes. Genes were separated based on position, with mitochrondrial and plastid genes assigned to "organellar" and all other genes assigned to "chromosomal." The dotted line represents chromosomal genes, and the solid line represents organellar genes

Figure S11

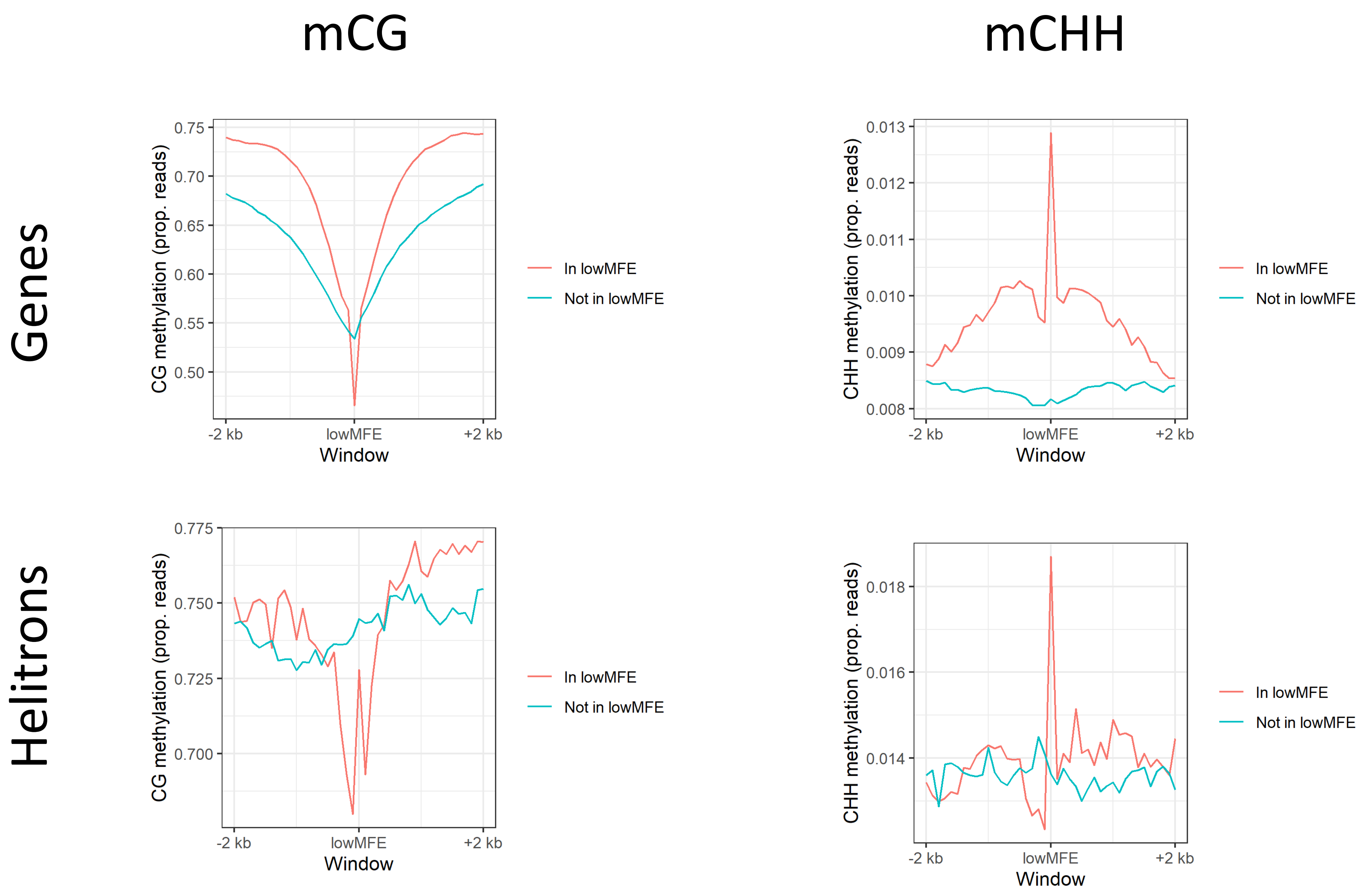

Figure S11. Methylation at lowMFE regions. The left column shows methylation in the CG context (mCG) and the right shows methylation in the CHH context (mCHH). Each row represents a different feature type. The red lines summarize the patterns of methylation in the hairpin (variable sizes, median = 23 nt) across all hairpins in a given feature type (e.g., all TIR hairpins, gene hairpins, etc.) and their flanking regions, divided into 40 nonoverlapping 100 bp windows. We assigned a control window to each hairpin in the dataset by choosing a random window of the same size as the hairpin within the same element. The blue line corresponds to methylation patterns around these randomized control loci.

Figure S11 (cont.)

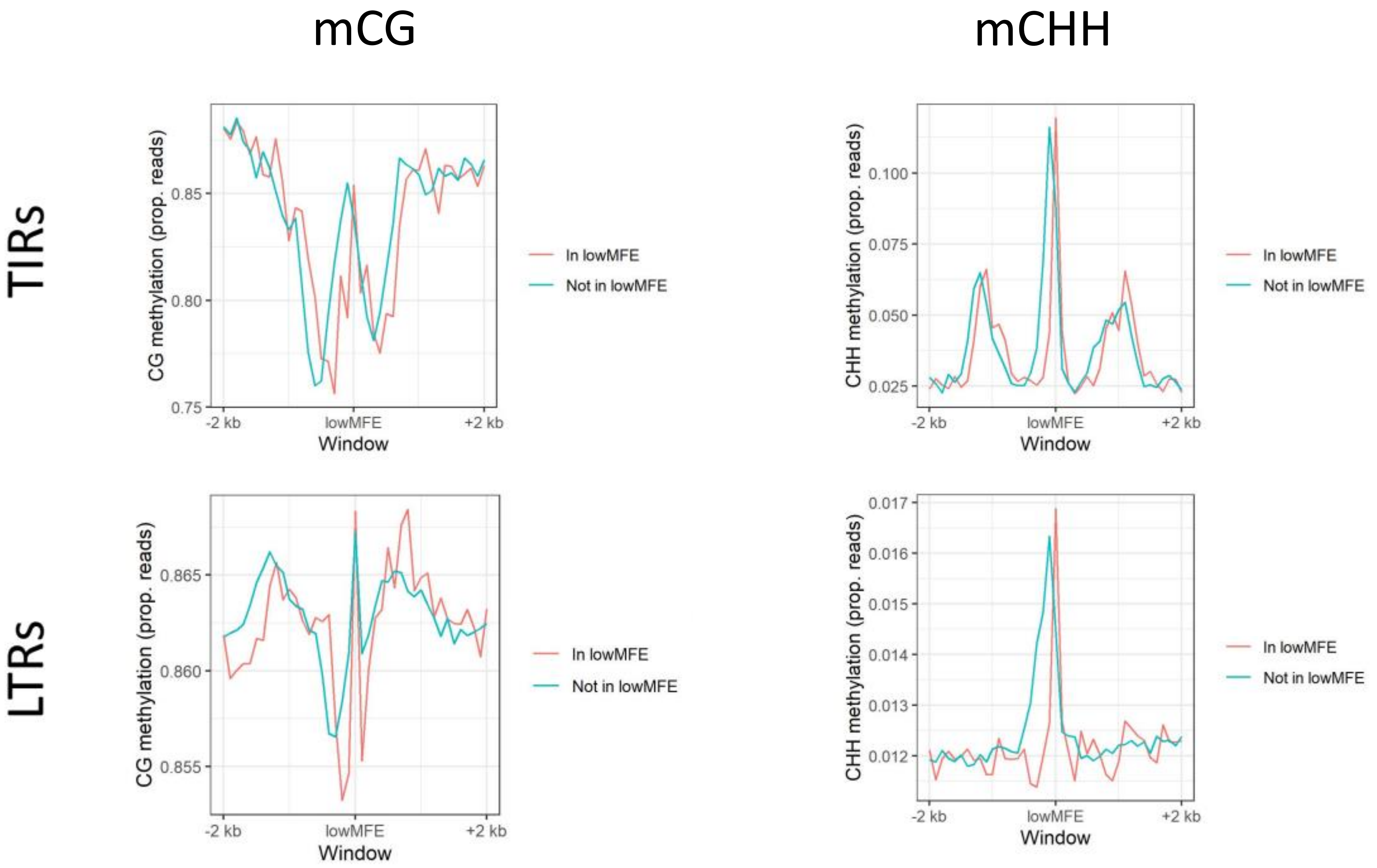

Figure S11. Methylation at lowMFE regions. The left column shows methylation in the CG context (mCG) and the right shows methylation in the CHH context (mCHH). Each row represents a different feature type. The red lines summarize the patterns of methylation in the hairpin (variable sizes, median = 23 nt) across all hairpins in a given feature type (e.g., all TIR hairpins, gene hairpins, etc.) and their flanking regions, divided into 40 nonoverlapping 100 bp windows. We assigned a control window to each hairpin in the dataset by choosing a random window of the same size as the hairpin within the same element. The blue line corresponds to methylation patterns around these randomized control loci.

Figure S12

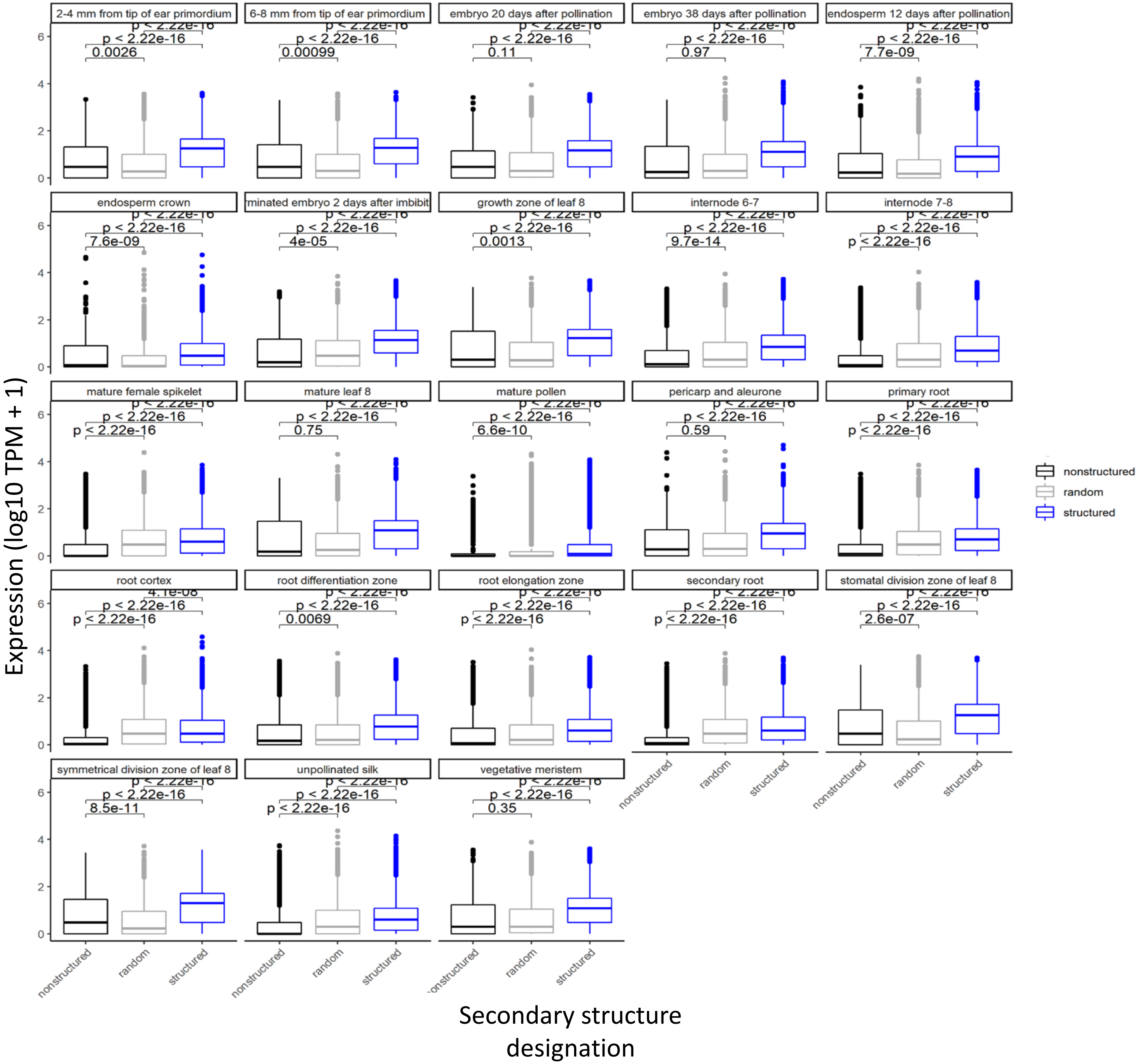

Figure S12. Expression between structured, random, and unstructured genes in 23 B73 tissues. “Structured” represent RF-structured genes, “random” represent genes with minMFE < -40 kcal/mol but which are not significantly different from the minMFE of 5 randomizations, and “unstructured” have minMFE > -40 kcal/mol.. Expression data are from Walley et al., 2016 and were downloaded from the ATLAS expression database (E-GEOD-50191). Boxplot central lines represent the median, and boxes represent the 25% and 75% quartiles.

Figure S13

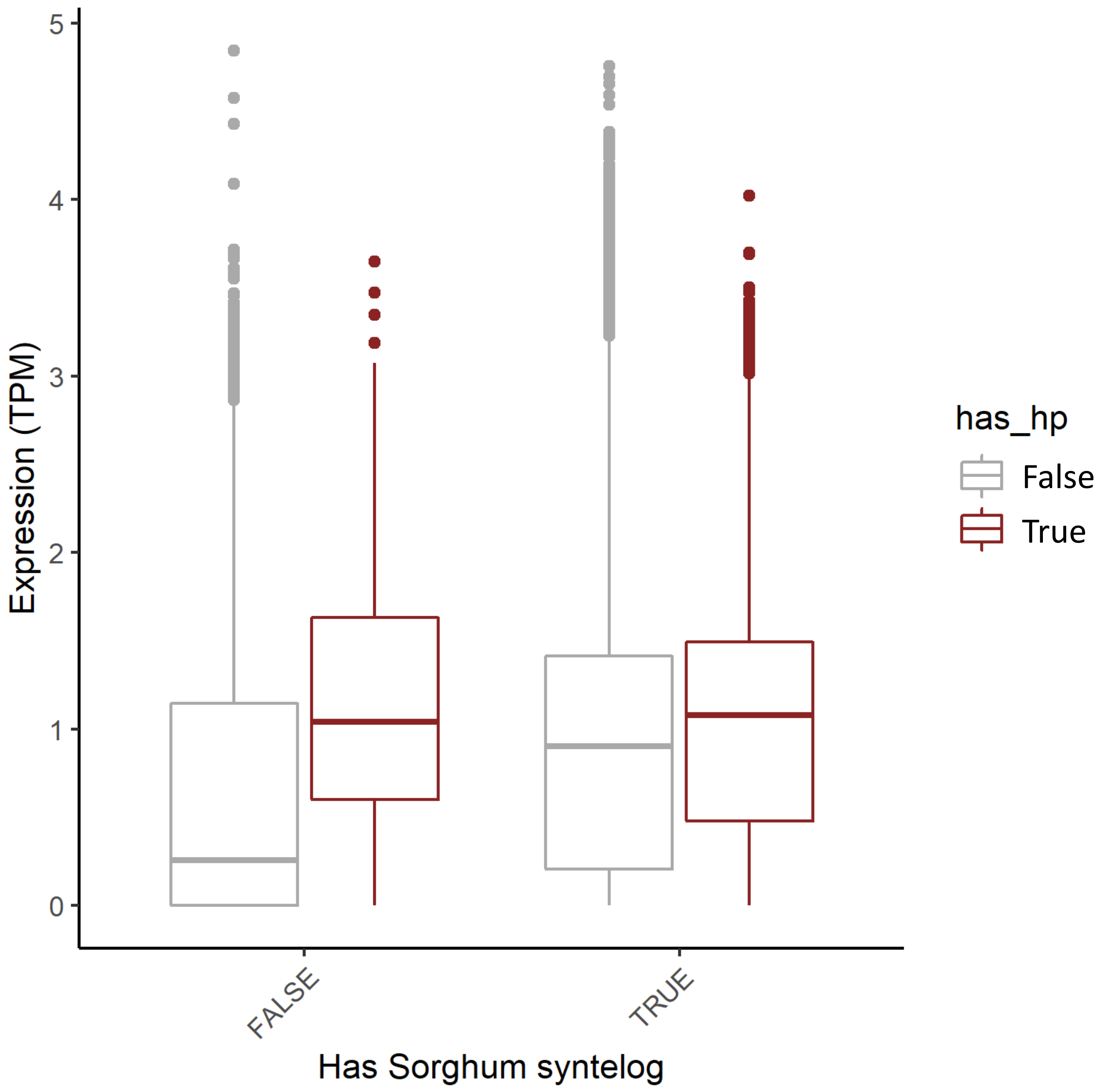

Figure S13. Expression between genes with and without LP-hairpins across 23 B73 tissues. Genes are divided into those with and without detectable *Sorghum bicolor* syntelogs (Muyle et al., 2021). Expression data are from Walley et al., 2016 and were downloaded from the ATLAS expression database (E-GEOD-50191). Boxplot central lines represent the median, and boxes represent the 25% and 75% quartiles.

Figure S14

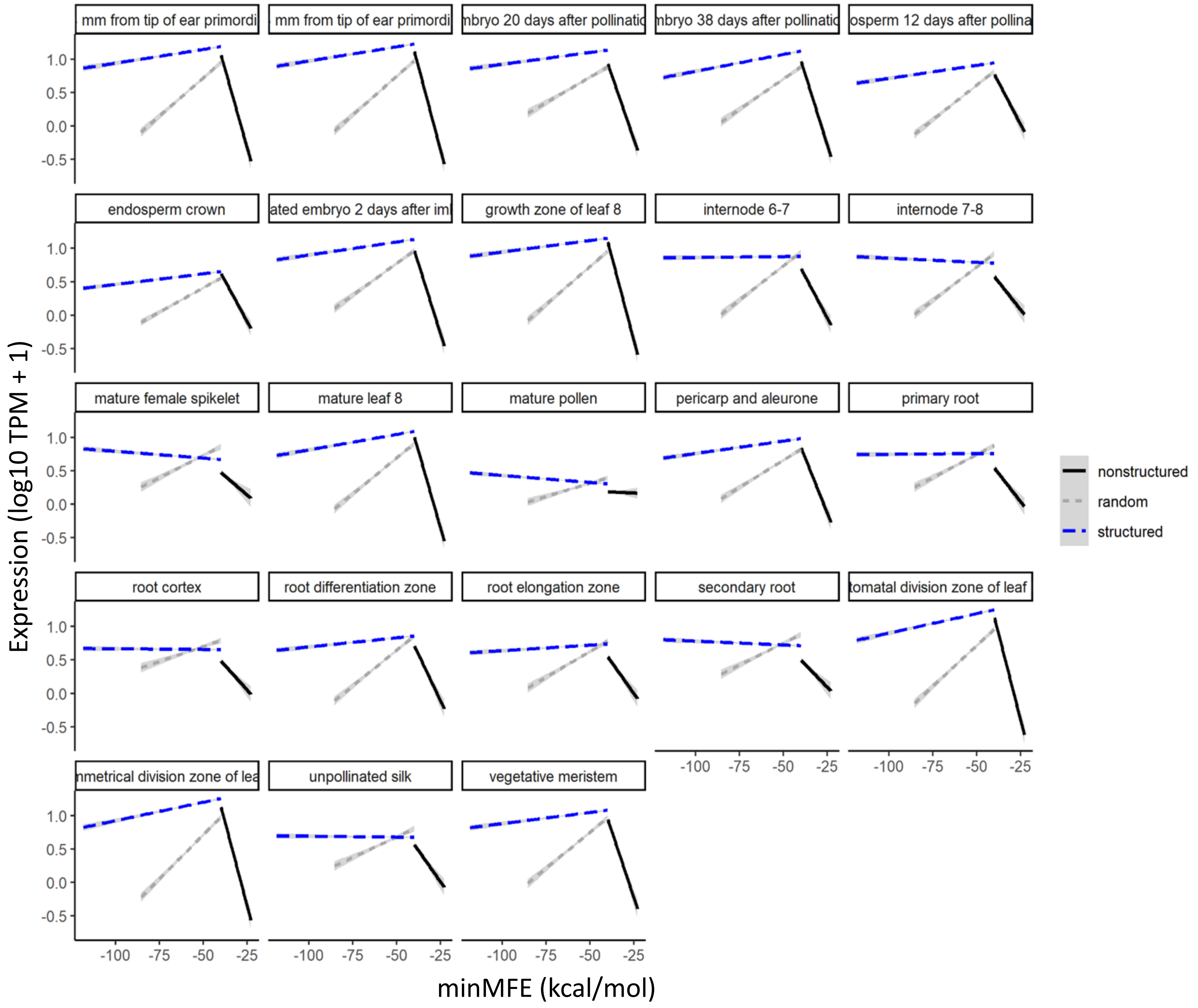

Figure S14. Expression as a function of minMFE in 23 B73 tissues. Expression is represented in log10 TPM+1, and structure designations are from the primary sequences in B73 (see **Fig S13**). Expression data are from Walley et al., 2016 and were downloaded from the ATLAS expression database (E-GEOD-50191).

Figure S15

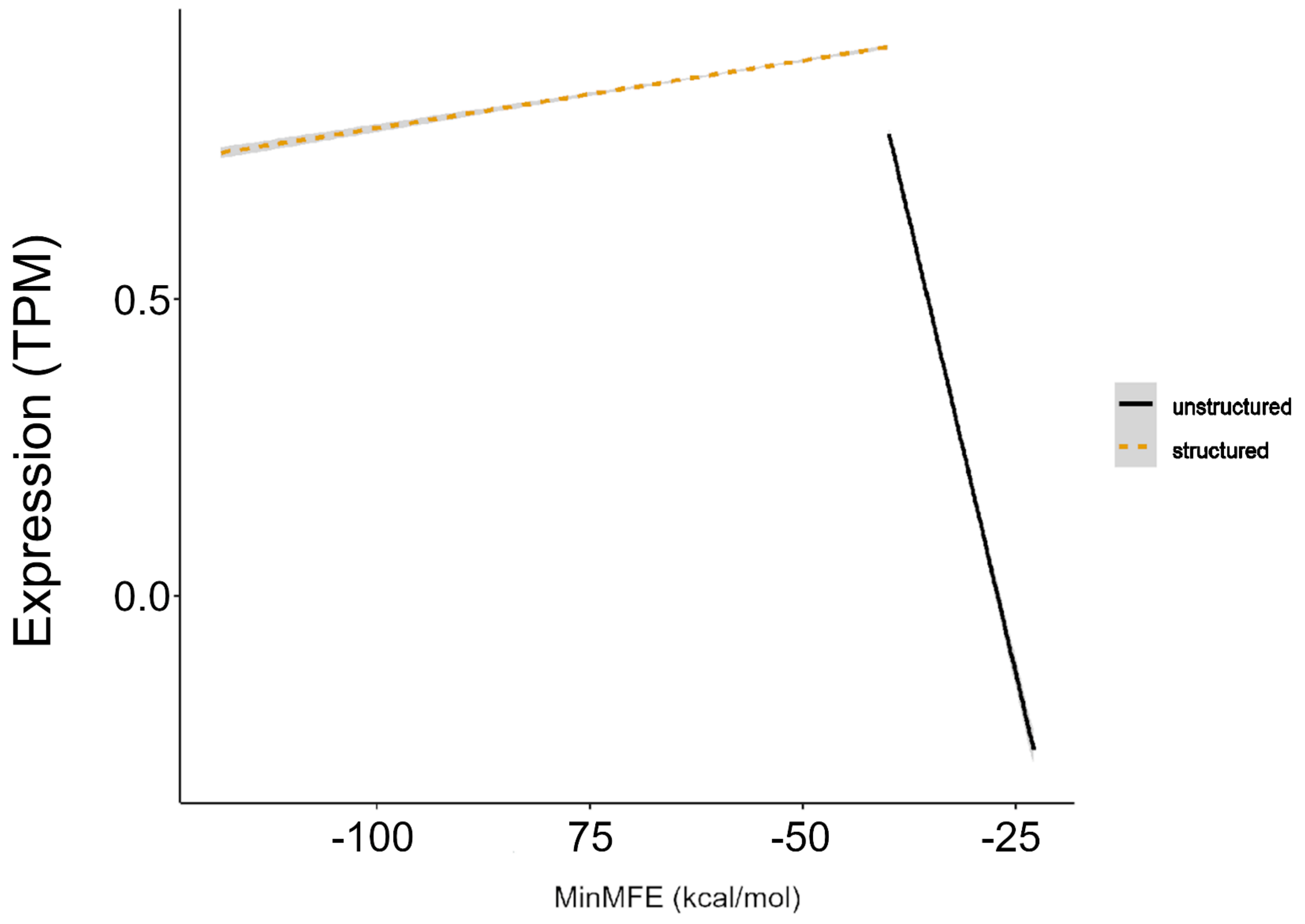

Figure S15. Expression as a function of minMFE for RF-structured and unstructured genes. In contrast to Fig. 4, all genes (with and without *Sorghum* syntelogs) are included.

Figure S16

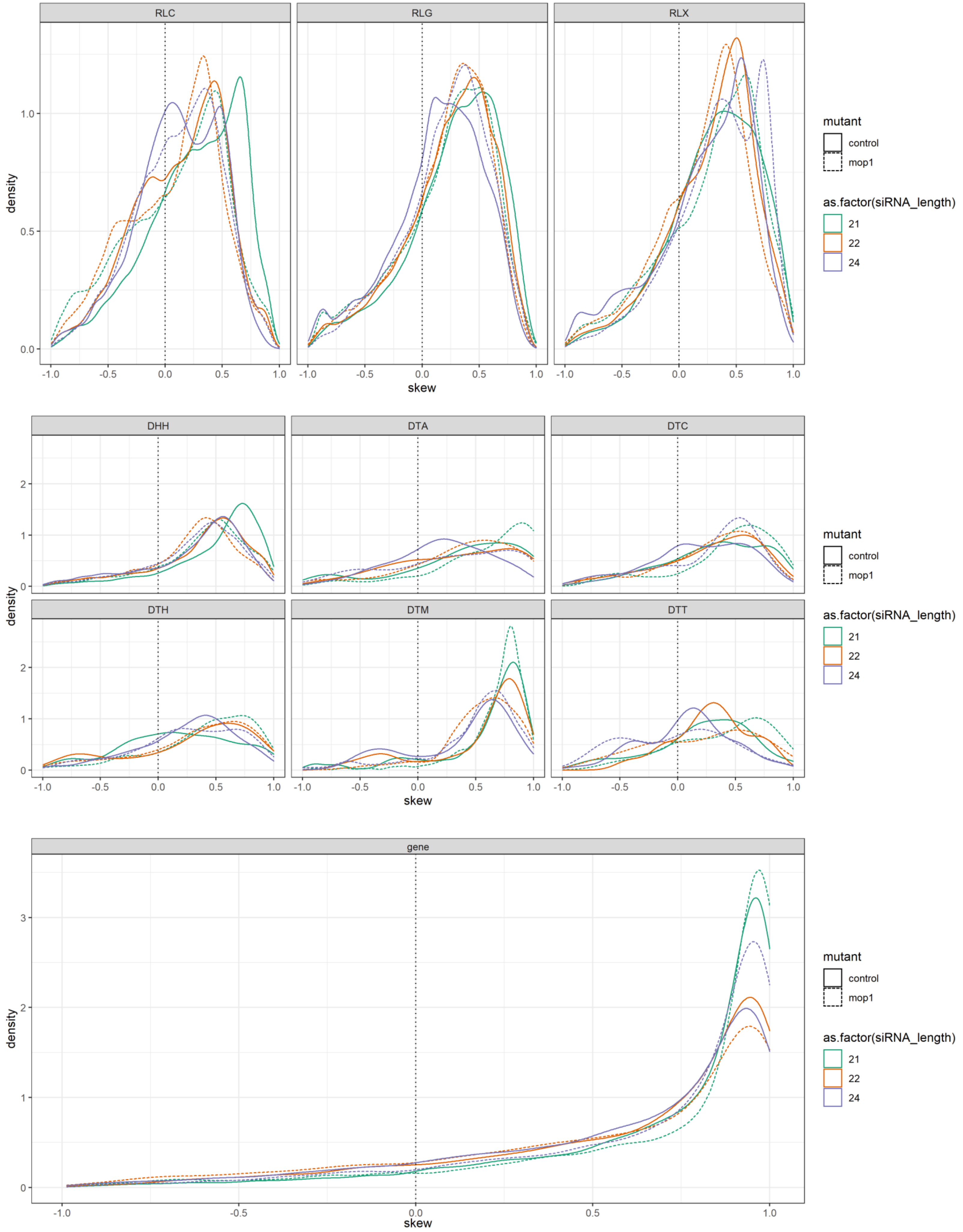

Figure S16. siRNA mapping skew for mutant vs wildtype control libraries in LP-hairpins. Solid lines represent control (WT) libraries, while dotted lines represent mop1 libraries. Skew was calculated as in Fig. 3.
